# Concept and study protocol of the process evaluation of a pragmatic randomized controlled trial to promote physical activity in outpatients with heterogeneous mental disorders—the ImPuls study

**DOI:** 10.1186/s13063-023-07331-y

**Published:** 2023-05-15

**Authors:** David Victor Fiedler, Stephanie Rosenstiel, Johanna-Marie Zeibig, Britta Seiffer, Jana Welkerling, Anna Katharina Frei, Thomas Studnitz, Julia Baur, Florian Helmhold, Andreas Ray, Eva Herzog, Keisuke Takano, Tristan Nakagawa, Saskia Kropp, Sebastian Franke, Stefan Peters, Anna Lena Flagmeier, Lena Zwanzleitner, Leonie Sundmacher, Ander Ramos-Murguialday, Martin Hautzinger, Thomas Ehring, Gorden Sudeck, Sebastian Wolf

**Affiliations:** 1 Faculty of Economics and Social Sciences, Institute of Sports Science, Department of Education & Health Research, University of Tübingen, Tübingen, Germany; 2 Faculty of Science, Psychological Institute, Department of Clinical Psychology and Psychotherapy, University of Tübingen, Tübingen, Germany; 3 Medical Faculty, Institute of Medical Psychology and Behavioral Neurobiology, University of Tübingen, Tübingen, Germany; 4 Department of Psychology, Clinical Psychology and Psychotherapy, LMU Munich, Munich, Germany; 5 Chair of Health Economics, Technical University Munich (TUM), Munich, Germany; 6 German Association for health-related Fitness and Exercise Therapy (German: DVGS), Hürth-Efferen, Germany; 7 AOK Baden-Württemberg, Stuttgart, Germany; 8 Techniker Krankenkasse, Hamburg, Germany

**Keywords:** Implementation research, Group-based exercise intervention, Behavior change techniques, MRC framework, Outpatient care, Mental disorders

## Abstract

**Background:**

Evidence suggests that patients suffering from different mental disorders benefit from exercise programs combined with behavior change techniques. Based on this evidence, we have developed an exercise program (ImPuls) specifically designed to provide an additional treatment option in the outpatient mental health care system. The implementation of such complex programs into the outpatient context requires research that goes beyond the evaluation of effectiveness, and includes process evaluation. So far, process evaluation related to exercise interventions has rarely been conducted. As part of a current pragmatic randomized controlled trial evaluating ImPuls treatment effects, we are therefore carrying out comprehensive process evaluation according to the Medical Research Council (MRC) framework. The central aim of our process evaluation is to support the findings of the ongoing randomized controlled trial.

**Methods:**

The process evaluation follows a mixed-methods approach. We collect quantitative data via online-questionnaires from patients, exercise therapists, referring healthcare professionals and managers of outpatient rehabilitative and medical care facilities before, during, and after the intervention. In addition, documentation data as well as data from the ImPuls smartphone application are collected. Quantitative data is complemented by qualitative interviews with exercise therapists as well as a focus-group interview with managers. Treatment fidelity will be assessed through the rating of video-recorded sessions. Quantitative data analysis includes descriptive as well as mediation and moderation analyses. Qualitative data will be analyzed via qualitative content analysis.

**Discussion:**

The results of our process evaluation will complement the evaluation of effectiveness and cost-effectiveness and will, for example, provide important information about mechanisms of impact, structural prerequisites, or provider qualification that may support the decision-making process of health policy stakeholders. It might contribute to paving the way for exercise programs like ImPuls to be made successively available for patients with heterogeneous mental disorders in the German outpatient mental health care system.

**Trial registration:**

The parent clinical study was registered in the German Clinical Trials Register (ID: DRKS00024152, registered 05/02/2021, https://drks.de/search/en/trial/DRKS00024152).

**Supplementary Information:**

The online version contains supplementary material available at 10.1186/s13063-023-07331-y.

## Introduction

Exercise can positively impact different mental disorders [1–7] and therefore can be seen as an additional treatment option for those patients. Despite the positive effects of exercise on mental health, patients with mental disorders have a lower likelihood of being sufficiently physically active  [8–10]. In fact, it is characteristic for this particular group of patients to find initiating and maintaining physical activity difficult [11]. Research focusing on patients with mental disorders stated that motivational and volitional strategies for behavior change can tackle this issue as these strategies may increase the level of physical activity [12] and thus play a crucial role in terms of sustainable behavior change [13]. Considering these specifics of patients with mental disorders (e.g., having motivational and volitional issues initiating and maintaining physical activity) [8, 11], the combination of behavior change techniques (BCT; [14]) and exercise as a structured intervention therefore appears highly promising in terms of initiating a sustainable exercise behavior change. Since outpatients have less supervision and contact to their therapists compared to patients in inpatient or rehabilitative mental health care settings, structured exercise interventions in combination with behavior change techniques to overcome general and disorder-specific barriers might be especially important within the outpatient mental health care setting. Indeed solely exercise on prescription (or on referral) for outpatients shows drop-out rates of nearly 80% [15], whereas structured exercise interventions in combination with BCTs for outpatients show lower dropout rates and stronger effects on mental health [16].

Therefore, ImPuls was developed and evaluated as a complex exercise program with respect to the Medical Research Council (MRC) framework, specifically designed to scrutinize an additional treatment option in the outpatient mental health care system in Germany [17]. It combines exercise (2 to 3 times per week running/fast walking at a moderate to vigorous intensity [≥ 64% max. heartrate] for 30 min, either with a standardized interval-based or an endurance method protocol; from week 2, patients can engage in an additional physical activity according to their personal preferences) with BCTs (regarding self-efficacy, goal setting, self-monitoring, affect regulation, formation of concrete exercise plans and coping planning) [18]. It has been successfully evaluated in terms of efficacy and acceptability in a feasibility study for outpatients waiting for psychotherapeutic treatment [19, 20]. A broader and more comprehensive pragmatic trial was needed to explore the extent to which the intervention also achieves its effect in a real-world setting (e.g., with exercise therapists working in the outpatient setting as intervention deliverers alongside their daily business; as an add-on to treatment as usual; with a realistic referral system) [21]. Therefore, it is now conducted in a pragmatic multi-site randomized controlled trial to investigate effectiveness and cost-effectiveness within the real-world outpatient setting [18].

The complexity of ImPuls (i.e., several interacting factors [e.g., BCTs and Exercise], involvement of different actors [exercise therapists, managers of outpatient rehabilitative and medical care facilities, patients] etc.) and the future need to implement the intervention into a comprehensive health service provision prompts the necessity of research beyond a pure evaluation of effectiveness, namely process evaluation. Thus, the ImPuls study is accompanied by a comprehensive process evaluation based on the MRC framework [22] and its complement [23], the former of which provides comprehensive and detailed guidance [24]. Using a mixed-methods approach may be particularly helpful to understand multiple perspectives, multiple types of causal processes and multiple types of outcomes which in turn are common aspects of implementation research [25].

Process evaluation in studies evaluating exercise interventions has been slowly emerging during the last decade [26–28]. However, process evaluation of exercise interventions offered to patients with mental disorders is rarely conducted. For example, a recent and very reputable meta-analysis on the effects of exercise on depression included 11 studies [2]. None of the included studies integrated a process evaluation. If process evaluations are conducted within this field, they are sparse and often limited. For example, one study with adolescents focused solely on selected aspects like adherence rate of the participants, acceptability, and feasibility [29], which means that only specific subcomponents of the MRC framework were taken into account. Other studies exclusively conducted qualitative interviews [30, 31], which ideally should be complemented by quantitative methods to provide an encompassing insight into the processes relevant for implementation [25]. Another study heeds the aforementioned deficiencies, yet apparently seems to omit investigation of interactions (e.g., between participants and the intervention/- deliverers) with regard to the MRC framework key component *mechanisms of impact* [32]. Given the lack of comprehensive process evaluations accompanying exercise programs for patients with heterogeneous mental disorders, the respective evidence for implementation conditions is weak. Consequentially, further research in this area is needed.

Our process evaluation focuses on the MRC’s three key components *implementation*, *context*, and *mechanisms of impact* and is conducted within the evaluation phase*.* In the area of *implementation*, we mainly focus on aspects of delivery (e.g., fidelity, quality of delivery) and recruitment (e.g., reach, coverage). With regard to *context* possibly affecting the aforementioned areas, we will mainly investigate the characteristics of outpatient rehabilitative and medical care facility managers as well as exercise therapists and of barriers and facilitators for the implementation of ImPuls (e.g., structural prerequisites). The interplay between patients, exercise therapists, and the program is one of our main topics when it comes to *mechanisms of impact*. This involves, for example, the extent to which patients and exercise therapists estimate ImPuls to be feasible, acceptable, appropriate, and relevant. Other main topics include patients’ integration of intervention core components (which may affect treatment effects) and changes within transdiagnostic processes (e.g., emotional regulation, repetitive negative thinking or perceived stress), which are utilized to explain treatment effects.

In sum, the main objectives of our process evaluation are a) to support the findings of the ongoing pragmatic randomized controlled trial by confirming that its effectiveness is truly attributable to the ImPuls intervention and b) to discover further crucial factors for the implementation of ImPuls into real-world outpatient mental health care settings.

The main research questions of the process evaluation are:

- 1) Implementation:
- a) To what extent did our actions empower exercise therapists (i.e., competence, acceptance) to deliver the intervention?
- b) To what extent do exercise therapists implement intervention components as intended (treatment fidelity) and what are reasons for its potential variance?
- c) Which strategies recruited the most patients and how valid were the referrals in terms of acquisition/inclusion?
- d) How do referring healthcare professionals rate the ImPuls intervention in terms of acceptability, appropriateness and feasibility?
- e) To what extent were all ImPuls sessions offered as planned and all telephone contacts made as scheduled?
- 2) Context:
- a) What barriers and facilitators did exercise therapists and managers experience concerning the implementation of the ImPuls intervention?
- 3) Mechanisms of Impact:
- a) To what extent do attitudes of exercise therapists towards the ImPuls intervention (e.g., acceptability, appropriateness) moderate the treatment effects?
- b) To what extent do patients’ integration of core components of the ImPuls intervention (e.g., amount of exercise, barrier management, goal-setting) as well as changes in respective individual behavioral determinants (e.g., action and coping plans; physical activity-related health competencies) mediate the treatment effects?
- c) To what extent do patients’ integration of motivational/volitional core components of the ImPuls intervention (e.g., barrier management, goal-setting, phone contacts) as well as changes in respective individual behavioral determinants (e.g., action and coping plans; physical activity-related health competencies) mediate its effect on their exercise adherence?
- d) To what extent do changes in patients’ transdiagnostic psychological processes (e.g., emotional regulation, repetitive negative thinking or perceived stress) mediate the treatment effects?

## Methods

Due to the aforementioned lack of process evaluations in the field of exercise interventions, we first provide a broad insight into the conceptualization of our process evaluation. Detailed information about the procedure of the process evaluation and the measurement tools are described within the section on data collection. Finally, the section on data analysis provides information about the operationalization of the main research questions and the planned analytical procedures.

### Concept of the ImPuls process evaluation

**Fig. 1:**
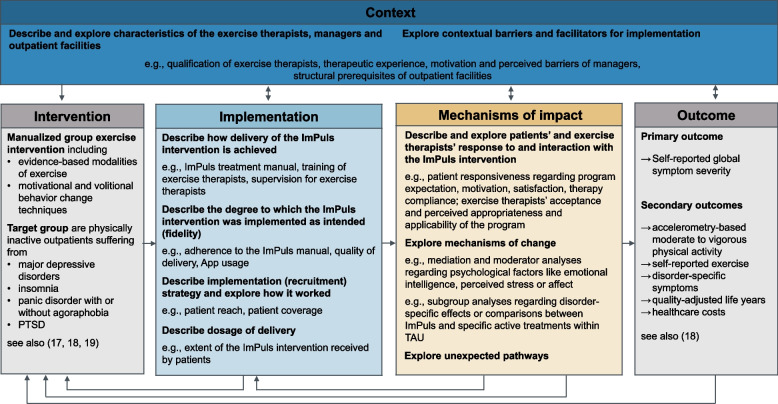
Overview of key functions of the ImPuls process evaluation adapted from the MRC Guidance [22]

We mainly focus on the key functions of process evaluation (see Fig. 1) by assessing and describing implementation, exploring and explaining mechanisms of impact and describing and exploring contextual factors, which are associated with why and how ImPuls (may) work in the real world setting [25, 33]. We use a mixed method approach to collect both qualitative and quantitative data from patients, exercise therapists, and managers of the outpatient rehabilitative and medical care facilities before, during, and after the intervention.

### Implementation

We will describe all actions implemented to ensure that the ImPuls intervention is delivered according to the treatment manual [22, 25] and will check to what extent the actions are appropriate and accepted in the target group of exercise therapists. Also, with certain requirements regarding their qualification [18] and with an ensuing ImPuls training, we want to ensure that exercise therapists are well qualified and prepared to conduct the ImPuls intervention in a high-quality fashion. To ensure that these standards were appropriate, accepted, and satisfactory to exercise therapists, we will evaluate their satisfaction with the ImPuls training and their perceived skill acquisition and self-efficacy in the application of the ImPuls intervention. Furthermore, we will describe and explore fidelity of the ImPuls intervention by rating adherence to the ImPuls manual and quality of delivery. However, there are indications that an adherence to the manual of about 80 to 100% can be seen as high fidelity, whereas a rate of 50% is determined as low fidelity [34]. A high fidelity may enhance treatment effects, especially with regard to increasing participants’ physical activity level [35]. Therefore, the first aim of our fidelity check is to test whether the high level of adherence (> 90%) that is aimed for in the context of the effectiveness trial is achieved. We will capture this by identifying whether and to what degree the exercise therapists are able to deliver the session-specific pre-defined core elements of the ImPuls intervention according to the treatment manual. In addition, we are also interested in the quality of delivery. For the ImPuls intervention this means the extent to which exercise therapists are able to show competent behavior related to the specifics of the ImPuls intervention that are supposedly at least partially new to them (e.g., questioning techniques and communication skills). Also, we will assess whether and how often the exercise therapists use the web-based ImPuls interface. In summary, our process evaluation intends to assess fidelity and examine reasons for its potential variance.

In addition, we will describe and explore to what extent the desired target group of patients with mental disorders is actually included in the study and/or therefore able to receive the ImPuls intervention [25]. For example, we are interested in whether or not we can reach enough patients within the respective age range, with the respective diagnoses, and in the intended regions with our recruitment strategies. As referring healthcare professionals differ in terms of experience with and access to this patient population, we will explore who has sent us patients and why. Finally, we will describe how much of the ImPuls intervention was delivered (dose) in-house by the exercise therapists in the outpatient rehabilitative and medical care facilities and thus potentially received by the patients [22, 36]. This includes ten supervised and three unsupervised ImPuls sessions during the supervised phase (weeks 0–4), the supporters meeting and phone calls once a week during the partially supervised phase 1 (weeks 5–12), and phone calls twice a month during the partially supervised phase 2 (weeks 13–24) carried out by the exercise therapists.

### Context

Against the background that the ImPuls intervention has never been conducted with the target group of patients with mental disorders in the real-world outpatient setting, we will describe and explore characteristics of exercise therapists, managers, and the outpatient rehabilitative and medical care facilities. These include, for example, qualification and therapeutic experience of exercise therapists, professional background, and motivation and perceived barriers of managers as well as structural characteristics of outpatient rehabilitative and medical care facilities such as premises, equipment, and staff. These contextual factors, external to the ImPuls intervention, can act as facilitators and/or barriers concerning the implementation and effectiveness of the program. For example, a therapist with only limited professional experience may generally find it difficult to offer exercise therapy in a group setting, which may also affect the quality and quantity of fidelity and, in turn, outcomes. Likewise, the conditions for exercise therapists when it comes to implementing the program in the facilities could differ. The conditions can be facilitating, for example, if there is strong support of managers who are interested in new treatment options. The conditions can also be obstructive, for instance, if difficulties arise when integrating the ImPuls intervention into pre-existing procedures, including time schedules, staff, and premises planning. These factors may have a positive or negative impact on the quantity and quality of implementation by the exercise therapist and may also affect their acceptance and their perception of the applicability of the program (see the “Mechanisms of impact” section).

### Mechanisms of impact

Patients take an active role in the whole intervention process by interacting with the ImPuls intervention and the exercise therapists [22]. We are interested in investigating their responses to the ImPuls intervention by, for instance, assessing their motivation and program expectation before engaging in the intervention and their satisfaction with both the treatment and their relationships to the exercise therapists after the ImPuls intervention. This also concerns the extent to which the patients use the ImPuls smartphone application (frequency) during the supervised and partially supervised phases and how they got on with the application. These reactions and interactions can have an impact on patient adherence (attendance and dropout rates). In this regard, patients will be considered as treatment dropouts (intended dose not reached), if they miss more than 4 supervised sessions in a row (≥ 40%, due to any reason) during the supervised phase. As the entire program builds upon the supervised period, attendance here is crucial for successful progression. Since ImPuls was developed as a transdiagnostic intervention for patients with heterogeneous mental disorders [17, 19], we assess already established transdiagnostic processes (e.g., emotional regulation, repetitive negative thinking or perceived stress).

As exercise therapists play a central role in the ImPuls intervention, we assume that it is equally important to examine their responses and interactions with the program and patients. Thus, for example, program-related attitudes (e.g., towards mental disorders, towards manualized interventions, expectation of program success), global self-efficacy, coping strategies, and motivation could have an impact on their relationship with patients (satisfaction with ImPuls group) or their quality of delivery. Likewise, perceptions of the appropriateness, relevance, and applicability of the ImPuls intervention may have an impact on their program acceptance and how they conduct the program. Positive and negative perceptions may in turn have an impact on the interaction between exercise therapists, patients, and the ImPuls intervention, which may explain a variation in outcomes. As outlined by different authors [22, 25, 37], qualitative interviews provide insights that allow for the exploration of unexpected mechanisms and pathways, such as facilitating or inhibiting factors in the realization of the program (e.g., technical or usability issues regarding the ImPuls smartphone application; certain processes in the daily therapy routine in the outpatient rehabilitative and medical care facilities).

### Data collection

Evaluation of effectiveness and process evaluation are conducted by separated research teams to avoid biasing the results. An external institution (Ludwig-Maximilians-Universität, Munich) conduct data management, storage, and analyses of quantitative data including questionnaires, documentations, video, and application data, while qualitative interview data is managed and processed by the process evaluation team.

In the following, we describe the procedure of the process evaluation and all measurements. SPIRIT reporting guidelines [38] are followed and a SPIRIT-protocol is provided in the additional files (see Additional file 1).

### Quantitative data

**Fig. 2:**
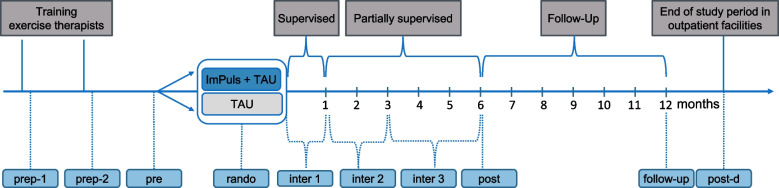
Design of the research project including all measurement time points of the process evaluation. Follow-up = 12 months after intervention start; Inter-1 = supervised ImPuls phase (weeks 1–4); Inter-2 = partially supervised ImPuls phase 1 (weeks 5–12); Inter-3 = partially supervised ImPuls phase 2 (weeks 13–24); Post = end of the intervention (week 24); Post-d = end of study period in the outpatient facility; Pre = prior to intervention start in the outpatient facility; Prep-1 = following the first training; Prep-2 = following the second training; Rando = after randomization, prior to intervention start in the outpatient facility. TAU (treatment as usual) is assessed only at pre, post, and follow-up. Assessments during inter 1 (weeks 1–4), inter 2 (weeks 5–12), and inter 3 (weeks 13–24) concern only exercise therapists and patients. Assessment frequency within inter-assessments punctually differs between participants (see Tables 1, 2, 3, and 4)

Exercise therapists, managers, and patients receive *online questionnaires* via the web-based data management system REDCap [39, 40] at different time points (see Fig. 2 and Table 1 (exercise therapists), Table 2 (managers), Table 3 (referring healthcare professionals), and Table 4 (patients). All participants receive an individual web-link via E-mail to access the online survey and have 2 weeks to complete it (except weekly assessments during inter phases 1–2, where patients have only 1 week to complete it). Reminders are automatically sent out 5 days after receiving the survey invitation (for weekly assessments: 3 days). We primarily use existing and already validated (in German) measures. If these were not available in German, we translated them using established backward translation procedures utilizing native speakers. In order to gain a more profound insight into the processes of the ImPuls intervention, in some measurements of the exercise therapists, managers, and physicians, we adapted the items specifically to ImPuls or developed items ourselves. Moreover, recruitment strategies allow for a variety of healthcare professionals (e.g., psychotherapists and primary care physicians) to refer patients to ImPuls. For this purpose, we ask them via online questionnaires about their opinion regarding exercise in combination with behavior change techniques as a new treatment option for patients with mental disorders.

**Table 1:** Measurements for exercise therapists at each time point (following SPIRIT template [38])

Assessment exercise therapists	Time point								
	Prep-1	Prep-2	Pre	Inter-1	Inter-2	Inter-3	Post	Follow-up	Post-d
Implementation									
Training—satisfaction [41] [modified]	x	x							
Training—perceived skill acquisition/self-efficacy (ImPuls intervention) [42–44] [adapted to Impuls]	x	x							
Supervision—participation rate				x			x		x
Supervision—satisfaction [41] [modified]				x			x		x
App—user frequency (app)				x	x	x			
Adherence (video)				x					
Quality of delivery (video)				x					
Dosage—amount sessions delivered (documentation)				x	x	x			
Mechanisms of impact									
Global self-efficacy ImPuls [44] [adapted to Impuls]			x						x
Attitudes towards mental disorders (OMS-HC; [45]) [translated + modified]			x						x
Attitudes towards manualized interventions (EBPAS-36D; [46])			x						
Motivation (SESSW; [47]) [modified]; (self-developed items)			x	x					x
Expectation of program success (PATHEV; [48])			x						
Program acceptance/satisfaction (B&F-A; [49]) [translated + modified]			x						x
Perceived barriers (B&F-A; [49]) [translated + modified]				x	x		x		
Satisfaction with the ImPuls group ([41], WAI-SR, [50]) [modified]				x			x		
Coping strategies (DPCCQ; [51]) [modified]				x			x		
App-usability (SUS; [52]) [translated + modified]				x	x		x		
App-functionality (MARS-G;[53])							x		
App-viability (self-developed)							x		
App-satisfaction (MARS-G;[53])				x	x		x		
Context									
Demographics	x								
Qualification (self-developed)	x								
Therapeutic experience (regarding exercise in group setting/with patients with mental disorders) (self-developed)	x								

Follow-up = once after 12 months; Inter-1 = once at the end of the supervised ImPuls phase (weeks 1–4); Inter-2 = once at the end of the partially supervised ImPuls phase 1 (weeks 5–12); Inter-3 = once at the end of the partially supervised ImPuls phase 2 (weeks 13–24); Post = after completion of the intervention (weeks 24–26, supervised and partially supervised) ImPuls phases; Post-d = end of study period in the outpatient facility; Pre = prior to intervention start in the outpatient facility; Prep-1 = following the first training; Prep-2 = following the second training; Rando = after randomization, prior to intervention start in the outpatient facility

*Abbreviations*: *B & F – A*, barriers and facilitators assessment instrument, *DPCCQ* Development of Psychotherapist Common Core questionnaire *EBPAS -36D*, evidence-based practice attitude scale (German version), *FPTM-40* therapy motivation questionnaire, *MARS-G* Mobile App Rating Scale (German version), *OMS-HC* opening minds scale for health-care providers, *PATHEV* measurement of therapy expectation and therapy evaluation of patients, *SESSW* scale for recording subjective school values, *SUS* system usability scale, *WAI-SR* Working Alliance Inventory—short revised (German version)

**Table 2:** Measurements for managers at each time point (following SPIRIT template [38])

Assessment managers	Time point								
	Prep-1	Prep-2	Pre	Inter-1	Inter-2	Inter-3	Post	Follow-up	Post-d
Context									
Qualification (self-developed)			x						
Structural characteristics of outpatient facilities [54–56] [modified]			x						
Motivation (self-developed)			x				x		x
Satisfaction [41, 57] [modified]							x		x
Perceived barriers (B&F-A; [49]) [translated + modified]			x				x		x

Follow-up = once after 12 months; Inter-1 = once at the end of the supervised ImPuls phase (weeks 1–4); Inter-2 = once at the end of the partially supervised ImPuls phase 1 (weeks 5–12); Inter-3 = once at the end of the partially supervised ImPuls phase 2 (weeks 13–24); Post = after completion of three ImPuls intervention groups in the outpatient facility; Post-d = end of study period in the outpatient facility; Pre = prior to intervention start in the outpatient facility; Prep-1 = following the first training; prep-2 = following the second training; Rando = after randomization, prior to intervention start in the outpatient facility

*Abbreviations*: *B & F – A*, barriers and facilitators assessment instrument

**Table 3:** Measurements for referring healthcare professionals at each time point (following SPIRIT template [38])

Assessment referrers	Time point								
	Prep-1	Prep-2	Pre	Inter-1	Inter-2	Inter-3	Post	Follow-up	Post-d
Implementation									
Professional background (self-developed)									x
Opinion on the new treatment option [58]; (self-developed items)									x
Physical activity level (EHIS-PAQ;[59]) [modified]									x

Follow-up = once after 12 months; Inter-1 = once at the end of the supervised ImPuls phase (weeks 1–4); Inter-2 = once at the end of the partially supervised ImPuls phase 1 (weeks 5–12); Inter-3 = once at the end of the partially supervised ImPuls phase 2 (weeks 13–24); Post = after completion of the intervention (weeks 24–26, supervised and partially supervised) ImPuls phases; Post-d = after end of study period in all outpatient facilities; Pre = prior to intervention start in the outpatient facility; Prep-1 = following the first training; Prep-2 = following the second training; Rando = after randomization, prior to intervention start in the outpatient facility

*Abbreviations*: *EHIS-PAQ* European Health Interview Survey—Physical Activity Questionnaire

**Table 4:** Measurements for patients at each time point (following SPIRIT template [38])

Assessment patients	Time point									
	Prep-1	Prep-2	Pre	Rando	Inter-1	Inter-2	Inter-3	Post	Follow-up	Post-d
Implementation										
Demographics			x							
Mechanisms of impact										
*Responses and interactions*^*a*^										
Motivation (FPTM-40; [60]) [modified]				x						
Expectation of program success (PATHEV; [48]) [modified]				x	x	x	x			
Satisfaction with the program (SSTS-R; [61]) [translated + adapted to Impuls]					x	x	x			
Therapeutic alliance (WAI-SR; [50])					x	x	x			
App										
User frequency (app)										
Usability (SUS; [52]) [translated + modified]					x	x	x			
Functionality (MARS-G; [53])							x			
Viability (self-developed)							x			
Satisfaction (MARS-G; [53])					x	x	x			
Attendance rate (documentation)					x					
Treatment dropouts (documentation)					x	x	x			
*Mechanisms of change*										
Emotional intelligence (TEIQue; [62])			x					x	x	
Emotion regulation (DERS; [63])			x					x	x	
Barrier management [64]			x					x	x	
Perceived stress (PSS; [65])			x					x	x	
Physical activity-related health competence (PAHCO; [66])			x					x	x	
Repetitive negative thinking (PTQ; [67])^b^			x		x	x	x	x	x	
Affect (state/trait) (PANAS; [68])^a, b^			x		x	x	x	x	x	
Affect/repetitive negative thinking during the program (App)^a, c^ (self-developed)					x	x	x			
Goal attainment during the program (app)^a, c^ (visual analog scale)					x	x	x			
Barrier management during the program (app)^a, c^ (visual analog scale)					x	x	x			
Frequency, intensity, time/duration and type of physical activity (FITT criteria, app)^a, c^					x	x	x			

Follow-up = once after 12 months; Inter-1 = once at the end of the supervised ImPuls phase (weeks 1–4); Inter-2 = once at the end of the partially supervised ImPuls phase 1 (weeks 5–12); Inter-3 = once at the end of the partially supervised ImPuls phase 2 (weeks 13–24); Post = after 6 months; Post-d = end of study period in the outpatient facility; Pre = before randomization, prior to intervention start in the outpatient facility; Prep-1 = following the first training; Prep-2 = following the second training; Rando = after randomization, prior to intervention start in the outpatient facility

^a^Intervention group only

^b^Weekly assessments (inter 1 = 4 times [weeks 1, 2, 3, and 4]); inter 2 = 8 times [weeks 5, 6, 7, 8, 9, 10, 11, and 12])/monthly assessment (inter 3 = 3 times [weeks 16, 20, and 24])

^c^Depending on patient’s usage of the smartphone application, data is collected continuously throughout the phases

*Abbreviations: DERS* difficulties in emotion regulation scale, *FITT* frequency, intensity, time/duration and type of physical activity, *FPTM-40* therapy motivation questionnaire, *MARS-G* Mobile App Rating Scale (German version), *PAHCO* physical activity-related health competence questionnaire, *PANAS* Positive and Negative Affect Schedule, *PATHEV* measurement of therapy expectation and therapy evaluation of patients, *PSS* perceived stress scale, *PTQ* perseverative thinking questionnaire, *SSTS-R* satisfaction with therapy and therapist scale (revised), *SUS* system usability scale, *TEIQue* trait emotional intelligence questionnaire, *WAI-SR* Working Alliance Inventory—short revised (German version)

Data of the *ImPuls smartphone application* was collected continuously during the supervised and partially supervised phase (inter 1–3; see Fig. 2 and Table 4). It will show the extent to which patients use the ImPuls smartphone application (frequency) in general as well as regarding different application functions including goal setting, barrier management, and training plans (patients’ integration of core components). Repetitive negative thinking and valence of affect are measured with a self-developed self-assessment manikin prior to and after each supervised and unsupervised exercise session over the entire intervention period. We also collect data from the *web-based ImPuls interface* accompanying the ImPuls smartphone application to record the extent to which exercise therapists have used the tool to review patients’ shared information during supervised and partially-supervised phases.

We receive *documentation data* from the exercise therapists and from the project staff. Exercise therapists are required to document whether all scheduled in-house sessions (supervised phase) and phone calls (partially supervised phase) were offered or completed (dose). As part of the recruitment process, project staff document how patients became aware of the project and how patients were distributed among outpatient rehabilitative and medical care facilities as well as patient dropouts and their reasons before and during the study. Recruitment strategies include flyers and posters at the offices of the participating outpatient rehabilitative and medical care facilities, primary care physicians, psychotherapists, and physiotherapists as well as a direct approach by health insurers involved in the project. We also disseminated information about the ImPuls intervention through self-help groups, daily newspapers, magazines, student mailing lists, and social media.

### Qualitative data

A *guided semi-structured interview* [69] was developed with respect to aspects of the MRC framework (e.g., acceptability, fidelity/delivery), empirical considerations prior to the intervention, and questions that arose over the course of the intervention (e.g., in conversations with exercise therapists). During the interviews, we ask exercise therapists about their experiences with patients in the ImPuls groups they conducted, their opinions about the program content regarding motivational and volitional BCTs and exercise, the perceived applicability of the program (regarding target group, general conditions in the outpatient rehabilitative and medical care facilities, ImPuls smartphone application and their own qualification), and their opinion on a possible long-term implementation of the ImPuls intervention. Possible reports of specific difficulties in implementing the program can provide us with information on why they may have had to deviate from the manual (adherence). Additionally, it can inform us regarding the areas in which they should have received more training. In summary, the interview focuses on facilitators and barriers for the implementation of the ImPuls intervention from exercise therapists’ perspectives. Interviews are conducted face to face by researchers of the process evaluation team with 20 exercise therapists, who all conducted at least one ImPuls group. The interviews have an estimated average duration of 50 min.

A *focus group interview* was developed analogous to the procedure mentioned above. It is supposed to provide an in-depth insight into managers’ perspectives concerning the perceived barriers and facilitators regarding the feasibility of the ImPuls intervention in the outpatient setting and its possible long-term implementation in the future. The focus group interview is conducted with 10 managers and is estimated to last 120 min.

Face to face interviews as well as the focus group interview are conducted once there are no further ImPuls groups in the respective outpatient rehabilitative and medical care facility. All interviews will be audio-recorded. Subsequently, the audio-records will be saved on a secured network drive of the University of Tübingen and transcribed verbatim by research assistants. All mentions of personal data will be masked during the transcription.

To assess *fidelity*, we recorded all 10 in-house sessions of each ImPuls group that have been conducted by the exercise therapists (except outdoor running activity) on video. We will randomly select one video for each group out of the eight core sessions to evaluate. This corresponds to 12.5% of all core sessions and 10% of all recorded sessions. Randomization will be done by an independent person who creates a randomization list using the software R version 4.1.2 [70]. We have already developed separate rating forms for each ImPuls session. Research assistants of the evaluation team will rate the sessions with regard to adherence to the treatment manual and quality of delivery. Raters will be trained in understanding the ImPuls manual and central features (setting S.M.A.R.T. goals, implement coping plans, execution of 30 minutes of exercise, discussion about perceived exertion, planning of preferred individual exercise training plans). Adherence to the treatment manual will be assessed by rating with “yes” (presence) or “no” (absence) as to whether pre-defined core elements of the ImPuls intervention have been delivered. An overall inter-rater reliability score will be calculated. Adherence will be calculated according to the overall amount of “yes”/”no” answers of these items. Subsequently, the percentage of all items answered with “yes” will be calculated in order to determine the final adherence percentage score. Quality of delivery will be rated once per session on a 4-point Likert scale ([1] totally agree–[4] totally disagree) using four items (exercise therapist listens actively, allows breaks and periods of reflection to take place [tolerates silence], includes all participants, takes statements of participants seriously). A sum score will be calculated.

### Data analyses

Empowerment of exercise therapists (research question 1a) will be analyzed descriptively (mean, standard deviation) with respect to their self-reports on the modified training evaluation scale [41], the (occupational-) self-efficacy scale [44], and concerning the frequency of supervision.

We will descriptively present the adherence score (mean, standard deviation) as well as the corresponding percentage to check whether the desired high level of adherence (> 90%) in the context of the effectiveness trial is achieved (research question 1b).

We will descriptively present the amount of all patients acquired by each strategy as well as the amount of all patients included in the study by each strategy. We will then check which recruitment strategy has the highest inclusion rate and present the corresponding percentage (research question 1c).

Referring healthcare professionals’ opinion on the new treatment option will be presented descriptively (mean, standard deviation) (research question 1d).

To provide information about the dose delivered (research question 1e), we will present documentation data of exercise therapists and the web-based ImPuls interface descriptively.

To assess barriers and facilitators (research question 2a), we will use quantitative data from questionnaires (barriers and facilitators assessment instrument (B&F-A; [49]), satisfaction scale [41, 57], as well as basic recommendations for outpatient facilities in Germany [54–56]). Results will be presented descriptively (mean, standard deviation) to provide an overview over contextual characteristics. This data will be further complemented with qualitative data from the interviews.

We will evaluate the interviews in a deductive-inductive process following the steps of a content-structuring qualitative content analysis [71]. First, two researchers will elaborate a preliminary coding frame for coding based on the interview guideline. Subsequently, 15% of all interviews will be independently coded by those researchers. Inter-coder-reliability analysis will then be performed to ensure that the elaborated coding frame is applicable. Potential discrepancies will be discussed to refine the coding frame in an iterative process. This process will be continued until both researchers agree that the categories are distinct and no new categories need to be added to the coding frame. Afterwards, the remaining interviews will be coded. Coding and analysis will be done by using the software MAXQDA 2022 [72]. In a final step, the statements from all interviews will be summarized category by category and used to supplement the quantitative data.

We will conduct mediation and moderation analyses as well as subgroup analyses to gain deeper insight into the impact of the program (mechanisms of change). We will check whether exercise therapists’ attitude towards mental disorders (opening minds scale for health-care providers (OMS-HC; [45])), evidence-based practice (evidence-based practice attitude scale—German version (EBPAS-36D; [46])), and the program (measurement of therapy expectation and therapy evaluation of patients (PATHEV; [48]), B&F-A) affects treatment effects (research question 3a). In addition, patients’ application data as well as changes in respective individual behavioral determinants (e.g., action and coping plans; physical activity-related health competencies (PAHCO; [66])) will be used to determine the extent to which core components of the intervention will have been used and how this affects treatment effects (research question 3b). Further analysis will be done to determine the extent to which motivational/volitional core components of the ImPuls intervention (application data [barrier management, goal-setting], documentation data [phone contacts]) as well as changes in respective individual behavioral determinants (e.g., action and coping plans; [PAHCO]) affect patients’ exercise adherence (research question 3c). Finally, we want to explore whether psychological processes such as emotional intelligence (trait emotional intelligence questionnaire (TEIQue; [62])), emotional regulation (difficulties in emotion regulation scale (DERS; [63])), repetitive negative thinking (perseverative thinking questionnaire (PTQ; [67])), or perceived stress (perceived stress scale (PSS; [65])) mediate the treatment effect on global symptom severity (research question 3d).

## Discussion

The aim of the present paper is to describe the planned process evaluation that will be conducted as part of the ongoing ImPuls effectiveness and cost-effectiveness trial [18]. Results of the process evaluation will be crucial for the future implementation of an exercise intervention in combination with BCTs for patients with mental disorders in the outpatient mental health care system (in Germany). The focus of our process evaluation, guided by the MRC framework [22], is to describe how we proceed by considering questions regarding implementation, mechanisms of impact, and context. However, the key functions should not be considered separately from one another but rather as related to each other. This allows for mechanisms of impact to be matched with implementation data, as certain mechanisms may be more effective when fidelity is higher [22].

The individual context of the outpatient rehabilitative and medical care facilities, managers, and exercise therapists could in turn influence both implementation and mechanisms of impact.

On the one hand, our approach has the purpose of explaining causal mechanisms as well as differences in outcomes. On the other hand, by exploring differences and variability in implementation, context, and mechanisms of impact, we aim to identify factors that may be barriers or facilitators to long-term implementation of the program in the outpatient mental health care system.

Especially a fidelity score of ≥ 90% might be very challenging to reach for the exercise therapists with respect to working with patients with heterogeneous mental disorders potentially experiencing mood swings, relapses, or even adverse events during sessions. However, our pilot study has proven the feasibility of ImPuls and a high fidelity score ensures that the ImPuls program is delivered as intended, which in turn can also be considered a quality criterion [34, 73]. As the ImPuls manual was developed intentionally with a moderate degree of standardization, it leaves exercise therapists the leeway to address the individual needs of patients, if necessary. In this way, both requirements could be met.

With regard to the recent addition to the MRC framework [23], the ImPuls intervention has already gone through the development and feasibility phases and is currently at the stage of evaluation, considering questions of implementation. It is planned that potentially successful results in effectiveness and cost-effectiveness as well as the findings of the process evaluation will be presented to health policy stakeholders (e.g., federal joint committee, health insurances, professional associations [German Association for health-related Fitness and Exercise Therapy (Deutscher Verband für Gesundheitssport & Sporttherapie e.V.; DVGS], physicians, psychotherapists]) in order to subsequently move one step further in the direction of systematically transferring research findings about exercise interventions like ImPuls into routine practice [74, 75]. This may include information about the necessary qualification of intervention deliverers or prerequisites in terms of the structural quality of outpatient rehabilitative and medical care facilities. Ideally, the findings of our process evaluation regarding implementation outcomes (e.g., fidelity, coverage, acceptability, appropriateness [25], key functions of the program/key mechanism of change and contextual factors) will provide a solid base for this purpose.

There are some limitations to our process evaluation that have to be considered. First, a few of the scales we use in our questionnaires are modified versions, for example, the Working Alliance Inventory [50] or instruments developed for this project (e.g., motivation of exercise therapists to participate in the ImPuls project), and therefore might be valid only to a limited extent. However, this enables us to tailor the items exactly to the characteristics of the ImPuls intervention and seems adequate as there are, to the best of our knowledge, no validated scales available that capture precisely the constructs we are aiming for. As there are no valid rating sheets for assessing fidelity in videotaped intervention sessions, we had to develop our own. In doing so, we were guided by approaches of other research in the field of cognitive behavioral therapy [76, 77] as well as by experience from our own projects [78]. Although carefully developed, our rating sheets may have a constrained validity. However, this engagement enabled us to rate the recorded video material in terms of fidelity.

It should also be noted that the generalizability of our study is limited. We are conducting ImPuls in 10 different outpatient rehabilitation and medical care facilities in Baden-Württemberg, a specific federal state of Germany. The findings therefore may be valid on a regional level. Since prevalence of mental disorders as well as the psychological and psychiatric treatment situation do not differ significantly between the federal states of Germany [79] and a nationwide uniform training of the exercise therapists is ensured by the DVGS, findings may be generalizable on a national level. However, it might be challenging to generalize the results to an international level, where the outpatient mental health care systems might have different underlying (political) structures or the financial viability of exercise as a treatment may differ. Thus, using the MRC framework may be beneficial to structure the findings in a way that may enhance the transferability to an international level.

Nevertheless, our study also shows clear strengths. We conduct an extensive process evaluation of the delivery of an exercise intervention which includes BCTs for patients with mental disorders. In doing so, we aim to address the mentioned lack of process evaluations and further contribute to implementation research by particularly focusing on the outpatient mental health care setting. As recommended [22], the researchers involved in the process evaluation are separated from the outcome evaluation team to reduce potential biases. Another strength is the inclusion of different perspectives (e.g., of exercise therapists, managers, researchers), which might facilitate informing the various stakeholders involved and thus possibly bring about a change in the provision of physical activity programs in the health care system for patients with mental disorders. From the beginning, we worked on building up good relationships—especially with the exercise therapists as well as the managers—in order to obtain trustworthy data. This is in line with recent MRC guidance, which emphasizes the engagement of relevant stakeholders enabling the delivery of solutions for real world practice [23]. In addition our mixed-methods approach provides an encompassing insight into the processes relevant for implementation [25].

To sum up, with our study, we set out to contribute to improvements in the current outpatient mental health care situation for patients with heterogeneous mental disorders. In addition, we may also potentially advance the evidence base concerning the impact of exercise interventions in combination with BCTs in the outpatient mental health care setting. The results of our process evaluation will ideally provide substantial information that may support the decision-making-process of health policy stakeholders and thereby pave the way for exercise programs like ImPuls to be made widely available in routine healthcare for patients with heterogeneous mental disorders in the (German) outpatient mental health care system.

Lastly, it should be mentioned that despite the positive effects of exercise on mental health, patients with mental disorders have a lower likelihood of being sufficiently physically active [8–10]. In this context, sufficiently physically active means 150 min per week of moderate-intensity aerobic physical activity or 75 min per week of vigorous-intensity aerobic physical activity according to the current German national recommendations concerning physical activity related to mental and physical beneficial effects for adults with a chronic disease (e.g., depression) [80]. However, it is also stated that even a lower level of activity can already have positive effects for this target group [2, 4, 19, 20, 81, 82]. In addition, concerning preventive effects of physical activity regarding mental disorders, even half of the recommended dose for physical activity seems to be sufficient for a beneficial effect [83]. In conclusion, ImPuls incorporates current empirical findings about beneficial effects of physical activity for people with mental disorders which is in accordance with the dose for preventive and curative effects regarding mental disorders.

## Trial status

Recruitment of the outpatient rehabilitation and medical care facilities and exercise therapists started in the beginning of 2020. The recruitment of patients began in January 2021, while the first groups were randomized in April 2021. Randomization lasted until 31 May 2022. Protocol version number: 1.0, date of first submission: 27 May 2022. Revised protocol version number: 2.0, date 27 January 2023. Revised protocol version number: 2.1, date 04 April 2023. Revised protocol version number: 2.2, date 18 April 2023.

## Supplementary Information

**Additional file 1.**

## Data Availability

Individual participant (patients, exercise therapists, managers) data that underlie the results reported in this article will be published after deidentification. Documents that will be shared further are as follows: study protocol, statistical analysis plan, analytic code, aggregated individual study data. Routine/administrative data from health insurances will not be made available. Access to data will be provided for anyone legitimately interested in it. Analytic code and aggregated individual study data will be made available on an online repository immediately after publication (or within the peer review process). Participants give informed consent to publish their data after deidentification (except for the routine/administrative data from the health insurances).

## Abbreviations

BCT: Behavior change techniques
B&F – A: Barriers and facilitators assessment instrument
DERS: Difficulties in emotion regulation scale
DVGS: Deutscher Verband für Gesundheitssport & Sporttherapie e.V. (German Association for health-related Fitness and Exercise Therapy)
EBPAS-36D: Evidence-based practice attitude scale—German version
MRC: Medical Research Council
OMS-HC: Opening minds scale for health-care providers
PAHCO: Physical activity-related health competencies
PATHEV: Measurement of therapy expectation and therapy evaluation of patients
PTQ: Perseverative thinking questionnaire
PSS: Perceived stress scale
TEIQue: Trait emotional intelligence questionnaire
