# Supplementary material for: Concept and study protocol of the process evaluation of a pragmatic randomized controlled trial to promote physical activity in outpatients with heterogeneous mental disorders—the ImPuls study
Source: Trials. 2023 May 15;24:330. doi: 10.1186/s13063-023-07331-y (PMC10186678; doi:10.1186/s13063-023-07331-y)
Supplement: Supplementary file 1 — Additional file 1. [file 13063_2023_7331_MOESM1_ESM.docx]

Protocol of the following trial:

**Efficacy and Cost-Effectiveness of a Transdiagnostic Group-Based Exercise Intervention: Study Protocol for a Pragmatic Multi-Site Randomized Controlled Trial**

**Trial registration**: ID: DRKS00024152, 05/02/2021

**Issue date of the study protocol**: August 3rd, 2021

**Protocol Amendment Number**: 02

**Authors**:

Sebastian Wolf ^1,2,*^, Britta Seiffer ^1,2^, Johanna-Marie Zeibig ^1,2^, Jana Welkerling ^1,2^, Leonie Louisa Bauer ^1,2^, Anna-Katharina Frei ^1,2^, Thomas Studnitz ^1,2^, Stephanie Rosenstiel ^1^, David Victor Fiedler ^1^, Florian Helmhold ^3^, Andreas Ray ^3^, Eva Herzog ^4^, Keisuke Takano ^4^, Tristan Nakagawa ^4^, Saskia Kropp ^5^, Sebastian Franke ^5^, Stefan Peters ^6^, Nadja El-Kurd ^7^, Lena Zwanzleitner ^8^, Leonie Sundmacher ^5^, Ander Ramos-Murguialday ^3^, Martin Hautzinger ^2^, Gorden Sudeck ^1^ and Thomas Ehring ^4^

^1^ Faculty of Economics and Social Sciences, Institute of Sports Science, Department of Education & Health Research, University of Tuebingen, Tuebingen, Germany

^2^ Faculty of Science, Psychological Institute, Department of Clinical Psychology and Psychotherapy, University of Tuebingen, Tuebingen, Germany

^3^ Medical Faculty, Institute of Medical Psychology and Behavioral Neurobiology, University of Tuebingen, Tuebingen, Germany

^4^ Department of Psychology, Clinical Psychology and Psychotherapy, LMU Munich, Munich, Germany

^5^ Chair of Health Economics, Technical University Munich (TUM), Munich, Germany

^6^ German Association for health-related Fitness and Exercise Therapy (German: DVGS), Hürth-Efferen, Germany

^7^ AOK Baden-Wuerttemberg, Stuttgart, Germany

^8^ Techniker Krankenkasse, Hamburg, Germany

**Revision Chronology:**

| August 2021 | Original |
| --- | --- |
| March 2022 | 2^nd^ Version |
| January 2023 | 3^rd^ Version |

# Funding

This trial is fully funded by the German Innovation Fund of the Federal Joint Committee of Germany from September 2020 to February 2024 (01NVF19022). This funding source had no role in the design of this study and will not have any role during its execution, analyses, interpretation of the data, or decision to submit results

**Authors contributions**: S.W., J-M.Z., B.S., N.E-K., L.Z., L.S., A.R-M., M.H., G.S., T.E. contributed to the conception and the design of the study. Original draft preparation was done by S.W. All authors contributed to the drafting and revision of the final study protocol. S.W., J.-M.Z., B.S, J.W., L.B., A.-K.F. are responsible for study organization, recruitment and diagnostics, training of the exercise therapists and data management. S.R., D.-V.F. and G.S. are responsible for the process evaluation, development of treatment fidelity score, data management. F.H., A.R. and A.R.-M. are responsible for the app development and maintenance, S.P. is responsible for the recruitment of the study sites and the qualification of the exercise therapists, N.E.-K. and L.Z. are the representatives of the 2 health insurances, providing the routine data for the health economics analysis and support patient recruitment, E.H., K.T., T.N. and T.E. are responsible for data management, data handling, the randomization procedure, analysis of treatment fidelity and statistical analysis, S.K., S.F., L.S. are responsible for the health economics analysis. All authors have read and agreed to the published version of the manuscript.

**Trial Sponsor:**

University of Tuebingen

Dr. Sebastian Wolf

Gartenstraße 19

72074 Tuebingen

Deutschland

Tel.: +49 (0)7071 29-73613

Mail: [sebastian.wolf@uni-tuebingen.de](mailto:sebastian.wolf@uni-tuebingen.de)

**Organizational Structure and responsibilities:**

| **Responsible** | **Institution** | **Responsibility** |
| --- | --- | --- |
| Dr. Sebastian Wolf | Institute of Sports Science, Education and Health Research, Junior Research Group „Exercise and Mental Health“, Eberhard Karls University Tuebingen ^1^ | Project PI: Coordination, Administration, Recruiting, Diagnostics, training of therapists |
| Prof. Dr. Martin Hautzinger | Department Psychology, Clinical Psychology and Psychotherapy, Eberhard Karls University Tuebingen^2^ | Consulting, principal investigator |
| Prof. Dr. Gorden Sudeck | Institute of Sports Science, Education and Health Research, Eberhard Karls University Tuebingen ^1^ | supervision for exercise therapists, training of exercise therapists, process evaluation |
| Dr. Ander Ramos  Murguialday | Institute of Medical Psychology and Behavioral Neurobiology  Eberhard Karls University Tuebingen^3^ | App programming, testing and maintenance |
| Prof. Dr. Thomas Ehring, | Department Psychology, Clinical Psychology and Psychotherapy, Ludwig Maximilians University Munich (LMU) ^4^ | Randomization, Data Management, Data storage, Analysis of clinical data |
| Prof. Dr. Leonie Sundmacher | Health Economics, Technical University Munich (TUM)^5^ | Health economics, Trust center |
| Angelika Baldus  Stefan Peters | German Association of health-related Fitness and Exercise Therapy (German: DVGS) ^6^ | Recruitment of study sites, Qualification of exercise therapists |
| Anna Lena Flagmeier | AOK Baden-Württemberg ^7^ | Implementation of treatment structure in health insurance system, data management of routine data, patient recruitment |
| Lena Zwanzleitner | Techniker Krankenkasse  ^8^ | Implementation of treatment structure in health insurance system, data management of routine data, patient recruitment |

# Introduction

*Item 6a: Description of research question and justification for undertaking the trial, including summary of relevant studies (published and unpublished) examining benefits and harms for each intervention.*

Epidemiological data from 2019 suggests that 15.6 % of the German population suffered from any mental disorder in 2019 (point prevalence) (Global Burden of Disease Collaborative Network, 2020). Mental disorders result in a considerable burden of disease, for example accounting for 6.4 % of overall disability-adjusted life years (DALYS) assessed in the 2019 epidemiological survey (Global Burden of Disease Collaborative Network, 2020). From 2008 to 2018, the proportion of mental disorders among all causes of death increased from 2.2% to 6.1% (Boehm, 2021). The most prevalent disorders in Germany are anxiety disorders and trauma- and stress-related disorders (point prevalence: 7.1 %), major depressive disorders (point prevalence: 4.3 %), as well as insomnia (point prevalence: 4 %) (Global Burden of Disease Collaborative Network, 2020; Hajak, 2001). Notably, these disorders often occur comorbidly (Kessler et al., 2005) and share common underlying aetiological and even maintenance mechanisms, such as high perceived stress (Hall et al., 2007; Kendler et al., 1999; Moreno-Peral et al., 2014), low self-efficacy (Iancu et al., 2015; Maciejewski et al., 2000), sleep disturbances (Harvey, 2002), elevated anxiety sensitivity (Coles et al., 2015; Naragon-Gainey, 2010; Olatunji & Wolitzky-Taylor, 2009), or repetitive negative thinking (Harvey, 2002; Wahl et al., 2019).

Worldwide, 27.2% of the DALYS attributable to mental disorders can be explained by major depressive disorders and 16.3 % by anxiety disorders (Rehm & Shield, 2019). In 2015, health care costs in Germany caused by mental disorders amounted to 44.4 billion euros (Destatis [Statistisches Bundesamt], 2015). Of this, 8.7 billion euros can be attributed to major depressive disorders, 1.7 billion euros to phobic and other anxiety disorders, and 1 billion euros to insomnia. Mental disorders account for 13.1% of total costs and represent the second highest cost group after cardiovascular disorders (46.4 billion euros, 13.7% of total costs). Major depressive (RR = 2.63) and anxiety disorders (RR = 1.41) have also been shown to increase the risk of cardiovascular disease (De Hert et al., 2018). Besides direct costs (e.g., treatment costs), mental disorders cause indirect costs on the German job market. With 14.4 billion euros overall, mental disorders caused the second-highest lost production costs of all diagnosis groups in 2019 (Federal Institute for Occupational Safety and Health [Bundesanstalt für Arbeitsschutz und Arbeitsmedizin (BAuA)], 2021). They further caused 117.2 million days (16.5% of all days) of incapacity to work, which is the longest absences per sick leave of all disorders (Rabe-Menssen et al., 2021). Amongst mental disorders, major depressive disorders accounted for the most days of incapacity to work (33.9 million days), followed by trauma- and stress-related disorders (21.6 million days). Anxiety disorders accounted for 7.6 million days and insomnia for 0.5 million days.

Despite the severe negative impact of mental disorders, it is estimated that in Germany only 10% of all affected individuals receive evidence-based treatment and only 2.5% receive psychological treatment (Nübling et al., 2014). In addition, even those receiving psychological treatment often have to wait before treatment initiation; for example, 40 % of outpatients waited three to nine months to start psychotherapeutic treatment in German health care settings (Federal Chamber of Psychotherapists [Bundespsychotherapeutenkammer (BPtK)], 2021). Longer waiting times are associated with worsening and chronicity of symptoms and the development of comorbid conditions ((BPtK)], 2018). The high prevalence and severe burden of mental disorders in combination with the large gap between people in need for treatment and those actually receiving it (Patel, 2012) illustrates the need to develop alternative efficacious, effective and efficient treatments.

Exercise, defined as physical activity that is planned, structured and repeated, with the primary aim to improve or maintain physical fitness (Caspersen et al., 1985, p. 128), has revealed positive therapeutic effects for diverse mental disorders (Ashdown-Franks et al., 2020). Most of the studied exercise interventions include aerobic activities (i.e. running) or a combination of aerobic exercise with strengthening activities. Recent meta-analyses on major depressive disorders and insomnia have shown large effects for exercise that were comparable to those of psychological treatment and psychopharmacological treatment (Banno et al., 2018; Morres et al., 2018). A recent meta-analysis on PTSD (Rosenbaum et al., 2015) found small to moderate effects; however, in two of the four studies included, the intervention comprised yoga. Looking at more recent evidence from RCTs focusing on interventions including aerobic exercise, large treatment effects were found (Goldstein et al., 2018; Powers et al., 2015). For panic disorder (with and without agoraphobia), RCTs have revealed large effects on symptomatology with both, acute exercise and structured multi-week aerobic exercise programs (Broocks et al., 1998; Esquivel et al., 2008). In addition, moderate to large effects have been reported for exercise as an augmentation to TAU for major depressive disorders, panic disorder and PTSD (Gaudlitz et al., 2015; Legrand & Neff, 2016; Powers et al., 2015). Key components of exercise interventions that have shown optimal therapeutic efficacy among patients with major depressive disorders, insomnia, panic disorder with or without agoraphobia and PTSD (Aylett et al., 2018; Jacquart et al., 2019; Morres et al., 2018; Stubbs et al., 2018) include aerobic exercise at a minimum of moderate intensity (MVAE) either or a combination of MVAE with resistance training, conducted two to three times per week, for 10 weeks with a session duration of at least 30 minutes, partially supervised or non-supervised.

Exercise might not only be a promising efficacious treatment but also carry the advantage of being highly efficient, since it can be delivered in group settings with relatively short durations (Morres et al., 2018), can be offered to patients with heterogeneous and burdensome mental disorders, can be expected to show a low likelihood of adverse effects, comes at a relatively low cost, and is suited to reduce the risk for cardiovascular diseases that frequently occur comorbid with mental disorders (Fiuza-Luces et al., 2013; Naci & Ioannidis, 2015). Furthermore, exercise can be performed and continued independently without professional supervision or only remote supervision. However, individuals suffering from mental disorders often have difficulties to initiate and maintain a physically active lifestyle (Schuch et al., 2017), which may be related in part to deficiencies in motivation and exercise-related self-regulatory skills in this population (Kramer et al., 2014). Reassuringly, there is evidence showing that exercise adoption and maintenance are mediated by motivational and volitional aspects, such as intention strength, action planning and barrier management (Fuchs et al., 2012). A recent meta-analysis shows that especially self-efficacy in building intentions and action planning is crucial for sustained exercise behavior change (Zhang et al., 2019). One possible way to promote such motivational and volitional aspects are the application of behavior change techniques (BCTs) (Abraham et al., 2009; Michie et al., 2011).

The combination of behavior change techniques and exercise as a structured intervention appears highly promising in terms of initiating a sustainable exercise behavior change. Since outpatients have less supervision and contact to their therapists compared to patients in inpatient or rehabilitative mental health care settings, structured exercise interventions in combination with behavior change techniques to overcome general and disorder-specific barriers might be especially important within the outpatient mental health care setting. Indeed solely exercise on prescription (or on referral) for outpatients shows drop-out rates of nearly 80% (Crone et al., 2008), whereas structured exercise interventions in combination with BCTs for outpatients show lower dropout rates and stronger effects on mental health (Morres, et al., 2019).

Therefore, ImPuls was developed and evaluated as a complex exercise program with respect to the Medical Research Council (MRC) framework, specifically designed to scrutinize an additional treatment option in the outpatient mental health care system in Germany (Wolf et al., 2020). It has been successfully evaluated in terms of efficacy and acceptability in a feasibility study for outpatients waiting for psychotherapeutic treatment (Zeibig et al., 2023; Zeibig et al., 2021). A broader and more comprehensive pragmatic trial was needed to explore the extent to which the intervention also achieves its effect in a real-world setting (e.g., with exercise therapists working in the outpatient setting as intervention deliverers alongside their daily business; as an add-on to treatment as usual; with a realistic referral system) (Ford & Norrie, 2016). Therefore, it is now conducted in a pragmatic multi-site randomized controlled trial to investigate efficacy and cost-effectiveness within the real-world outpatient setting (Wolf et al., 2021).

The complexity of ImPuls (i.e., several interacting factors [e.g., BCTs and Exercise], involvement of different actors [exercise therapists, managers of outpatient rehabilitative and medical care facilities, patients] etc.) and the future need to implement the intervention into a comprehensive health service provision prompts the necessity of research beyond a pure evaluation of efficacy, namely process evaluation. Thus, the ImPuls study is accompanied by a comprehensive process evaluation based on the MRC framework (Moore et al., 2015) and its complement (Skivington et al., 2021), the former of which provides comprehensive and detailed guidance (Fynn et al., 2020). Using a mixed-methods approach may be particularly helpful to understand multiple perspectives, multiple types of causal processes and multiple types of outcomes which in turn are common aspects of implementation research (Peters et al., 2013).

Process evaluation in studies evaluating exercise interventions has been slowly emerging during the last decade (Ellard et al., 2014; van Dongen et al., 2018; Voorn et al., 2016). However, process evaluation of exercise interventions offered to patients with mental disorders is rarely conducted. For example, a recent and very reputable meta-analysis on the effects of exercise on depression included 11 studies (Morres, et al., 2019). None of the included studies integrated a process evaluation. If process evaluations are conducted within this field, they are sparse and often limited. For example, one study with adolescents focused solely on selected aspects like adherence rate of the participants, acceptability and feasibility (Jarbin et al., 2021), which means that only specific subcomponents of the MRC framework were taken into account. Other studies exclusively conducted qualitative interviews (Carr et al., 2021; Walburg et al., 2022), which ideally should be complemented by quantitative methods to provide an encompassing insight into the processes relevant for implementation (Peters et al., 2013). Another study heeds the aforementioned deficiencies, yet apparently seems to omit investigation of interactions (e.g., between participants and the intervention / - deliverers) with regard to the MRC framework key component mechanisms of impact (Farrand et al., 2014). Given the lack of comprehensive process evaluations accompanying exercise programs for patients with heterogeneous mental disorders, the respective evidence for implementation conditions is weak. Consequentially, further research in this area is needed.

*Item 6b: Explanation for choice of comparators.*

The TAU condition will be modelled to represent the typical treatment patients receive in the German outpatient health care system. Therefore, patients will not be actively provided with any treatment but patients are allowed to receive any intervention that is available to them. Any evidence-based treatment provided by the outpatient mental health care system will be recorded, i.e., any psychiatric/pharmacological or psychological/psychotherapeutic intervention. Interventions, delivery or dosage of the intervention can be changed and adapted during the course of the study.

*Item 7: Specific objectives or hypotheses.*

Despite the promising evidence for MVAE as an intervention for patients with mental disorders, exercise programs or professional exercise therapy are currently not provided as regular health services within the outpatient mental health care system in Germany. With the aim of combining the current evidence on the efficacy of MVAE and sustained exercise behavior change with specific demands of a real-world outpatient health care setting, ImPuls was developed as a manualized group exercise intervention (Wolf et al., 2020; Zeibig et al., 2021) for physically inactive outpatients suffering from major depressive disorders, insomnia, panic disorder with or without agoraphobia and PTSD. ImPuls integrates recent findings about the optimal modalities of exercise for therapeutic efficacy, such as optimal frequency, intensity, time/duration and type of exercise (FITT criteria) for the targeted disorders and evidence regarding sustainable behavior change by integrating behavior change techniques (BCTs). The components of this intervention are further tailored towards the specific needs of outpatients with mental disorders in the current German mental health care setting. Specific features are 1) the inclusion of a broad range of heterogenous diagnoses for which prior research has demonstrated therapeutic efficacy, 2) intervention delivery in group format, and 3) short duration (i.e., only 4 weeks of supervised MVAE sessions carried out inhouse in each study site). The aim of the current study is to investigate the efficacy and cost-effectiveness of implementing ImPuls within the outpatient mental health care setting in Baden-Württemberg, a representative state in South-West Germany. The following hypotheses will be tested:

1. Participants in the intervention condition, who have received ImPuls in addition to TAU, will show lower global symptom severity at post-treatment and follow-up assessments compared to a control condition with TAU only.

2. Overall costs in the intervention condition will represent a significant saving for the public health system compared to the control condition at post-treatment and follow-up assessments.

3a. The intervention will lead to significantly higher levels of MVAE at post-treatment and follow-up assessments compared to the control condition.

3b. The effect of condition on the reduction of the primary outcome global symptom severity will be mediated by an increase in MVAE.

4. Participants in the intervention condition will show lower disorder-specific symptoms (major depressive disorder, insomnia, panic disorder with or without agoraphobia and PTSD) compared to participants in the control condition at post-treatment and follow-up assessments

We will further assess, if participants in the intervention condition show more instances of clinically significant change compared to participants in the control condition at post-treatment and follow-up assessments (Additional analysis to Hypothesis 1 and 4)

The main objectives of our process evaluation are a) to support the findings of the ongoing pragmatic randomized controlled trial by confirming that its efficacy is truly attributable to the ImPuls intervention and b) to discover further crucial factors for the implementation of ImPuls into real-world outpatient mental health care settings.

The main research questions of the process evaluation are:

1. Implementation:
   1. To what extent did our actions empower exercise therapists (i.e., competence, acceptance) to deliver the intervention?
   2. To what extent do exercise therapists implement intervention components as intended (treatment fidelity) and what are reasons for its potential variance?
   3. Which strategies recruited the most patients and how valid were the referrals in terms of acquisition/inclusion?
   4. How do referring healthcare professionals rate the ImPuls intervention in terms of acceptability, appropriateness and feasibility?
   5. To what extent were all ImPuls sessions offered as planned and all telephone contacts made as scheduled?
2. Context:
   1. What barriers and facilitators did exercise therapists and managers experience concerning the implementation of the ImPuls intervention?
3. Mechanisms of Impact:
   1. To what extent do attitudes of exercise therapists towards the ImPuls intervention (e.g., acceptability, appropriateness) moderate the treatment effects?
   2. To what extent do patients’ integration of core components of the ImPuls intervention (e.g., amount of exercise, barrier management, goal-setting) as well as changes in respective individual behavioral determinants (e.g., action and coping plans; physical activity-related health competencies) mediate the treatment effects?
   3. To what extent do patients’ integration of motivational/volitional core components of the ImPuls intervention (e.g., barrier management, goal-setting, phone contacts) as well as changes in respective individual behavioral determinants (e.g., action and coping plans; physical activity-related health competencies) mediate its effect on their exercise adherence?
   4. To what extent do changes in patients' transdiagnostic psychological processes (e.g., emotional regulation, repetitive negative thinking or perceived stress) mediate the treatment effects?

*Item 8: Description of trial design including type of trial (e.g., parallel group, crossover, factorial, single group), allocation ratio, and framework (e.g., superiority, equivalence, non-inferiority, exploratory).*

The study will be led by researchers based at the University of Tuebingen in Germany, and will be conducted in 10 different study sites across Baden-Württemberg, a region in South-West Germany. The entire project will be conducted between September 2020 and February 2024. The study has been registered at the German Clinical Trial Register (ID: DRKS00024152, 05/02/2021) and has been approved by the local ethics committee for medical research at the University of Tuebingen (ID: 888/2020B01, 02/11/2020). A pragmatic multi-site block-randomized controlled trial with two treatment arms (ImPuls + TAU vs. TAU) and three points of assessment (pre, post, follow-up) will be conducted (see fig. 1). All outcomes will be included at all assessments. Study completion and reporting will be carried out in accordance with the Consolidated Standards of Reporting Trials (CONSORT) [52], the Template for Intervention Description and Replication (TIDieR) (Hoffmann et al., 2016) and the Consensus on Exercise Reporting Template (CERT) (Slade et al., 2016).

# Methods

## Study Setting

*Item 9: Description of study settings (e.g., community clinic, academic hospital) and list of countries where data will be collected. Reference to where list of study sites can be obtained.*

Data will be collected in Baden-Württemberg, a region south-west of Germany. Study sites are mostly big centers of psychosomatic, orthopedic or cardiological rehabilitation and one outpatient unit of physiotherapy. A list of all locations/study sites can be obtained in the registration: <https://www.drks.de/drks_web/setLocale_EN.do>

## Eligibility Criteria

*Item 10: Inclusion and exclusion criteria for participants. If applicable, eligibility criteria for study centres and individuals who will perform the interventions (e.g., surgeons, psychotherapists).*

**Participants.** Inclusion criteria are age between 18 and 65 years, membership of the AOK BW or TK, fluent in German, no medical contraindications for exercise, and diagnosed according to ICD-10 with at least one of the following disorders: depressive disorders (F32.1, F32.2, F33.1, F33.2), agoraphobia (F40.0, F40.01), panic disorder (F41.0), PTSD (F43.1) or insomnia (F51.0). Exclusion criteria included: Exercising of at least twice a week for at least 30 minutes each, continuously over a period of 6 weeks within the last 3 months before study diagnosis, sports-medical contraindication (medical consultation), acute mental and behavioral disorders due to psychotropic substances (F10.0, F10.2-F10.9; F11.0, F11.2-F11.9; F12. 0, F12.2-F12.9; F13.0, F13.2-F13.9; F14.0, F14.2-F14.9; F15.0, F15.2-F15.9; F16.0, F16.2-F16.9; F17.2-F17.9; F18.0, F18.2-F18.9; F19.0, F19.2-F19.9), acute eating disorders (F50); acute bipolar disorder (F31), acute schizophrenia (ICD-10 F20), acute suicidality.

**Exercise therapists/study therapists.** To carry out the intervention, exercise therapists are required to have one of the following academic or comparable basic qualifications as physical activity and exercise professionals with a training period of at least 3 years: academic degree in exercise or movement science with at least 10 ECTS practice and 20 ECTS theory (e.g. Magister, Bachelor, Master, Diploma Physical Education, Exercise Science, Exercise Physiology), non-academic technical college degree Exercise and Caring/Therapeutic Gymnastics with at least 21 semester hours per week, non-academic technical college degree „Physical Educator as liberal profession“, academic and non-academic degrees in physiotherapy. Moreover, a specific additional therapeutic qualification DVGS e.V. with 5 ECTS overall is required with the following content: Physical Activity-related Health Competence (2 ECTS), Basics in Health Science and Health Pedagogy (1 ECTS), Basics in Psychiatry, Psychosomatics and Addiction (1 ECTS), Affective Disorders (1 ECTS).

# Interventions

*Item 11a: Interventions for each group with sufficient detail to allow replication, including how and when they will be administered.*

**ImPuls.** The exercise intervention “ImPuls” (Wolf et al., 2020) will be delivered to groups consisting of 6 patients and will be divided into a supervised and partially-supervised period. BCTs, such as goal setting, self-monitoring, formation of concrete exercise plans and coping planning, will be integrated to promote sustained exercise behaviour change (Abraham et al., 2009; Geidl et al., 2012; Michie et al., 2011). The intervention structure and contents are displayed in Figure 3 and Table 1. Participants will receive ImPuls in addition to TAU.

***Supervised Period (weeks 0-4).*** Patients will participate in a combination of supervised MVAE sessions and group sessions with educative elements integrating BCTs (see table 1) in groups with a total duration of 120 minutes each session. Supervised MVAE will be provided twice a week and will consist of either running or fast walking. MVAE will last 30 minutes and participants can choose between a standardized interval-based or endurance method protocol. Both training methods will be conducted with at least moderate intensity, which is tracked by a heart rate monitor (SIGMA iD.FREE) combined with a chest strap (SIGMA R1 Bluetooth Duo Comfortex+) and the Borg Rating of Perceived Exertion (RPE) Scale (Borg, 1982). Moderate to vigorous intensity is defined as at least 64 % of maximum heart rate, subtracting age from 220 (Garber et al., 2011) and at least 13 points of the RPE Scale (Borg, 1982). The ImPuls smartphone application (“ImPuls-App”) developed specifically for ImPuls supports the participants and therapists during MVAE. In weeks 2, 3 and 4 patients will engage in additional 30-minutes MVAE, which is chosen based on their own interests and preferences. Therapists provide a list of MVAE highlights in each study site (i.e. offers in local sport clubs, yoga studio, gyms) which can be found and selected in the “ImPuls-App”.

### Partially Supervised Period (weeks 5-24). Participants will be asked to engage in 30-minutes non-supervised MVAE at least twice a week. Regular MVAE will be planned through specific training schedules and accompanied by activity diaries, self-monitoring of goals and volitional strategies and weekly (weeks 5-12)/biweekly (weeks 13-24) phone calls with the exercise therapist, intending to maintain motivation, volition, and adherence to exercise. Training plans and all documentations will be executed and coordinated via the “ImPuls-App”. Information will be shared with the therapists in advance prior to the phone calls. A session for patient’s supporters (e.g., friends, partner) will be scheduled in Week 5 to inform them about the possibilities to support the participants in transforming their intentions into action.

**
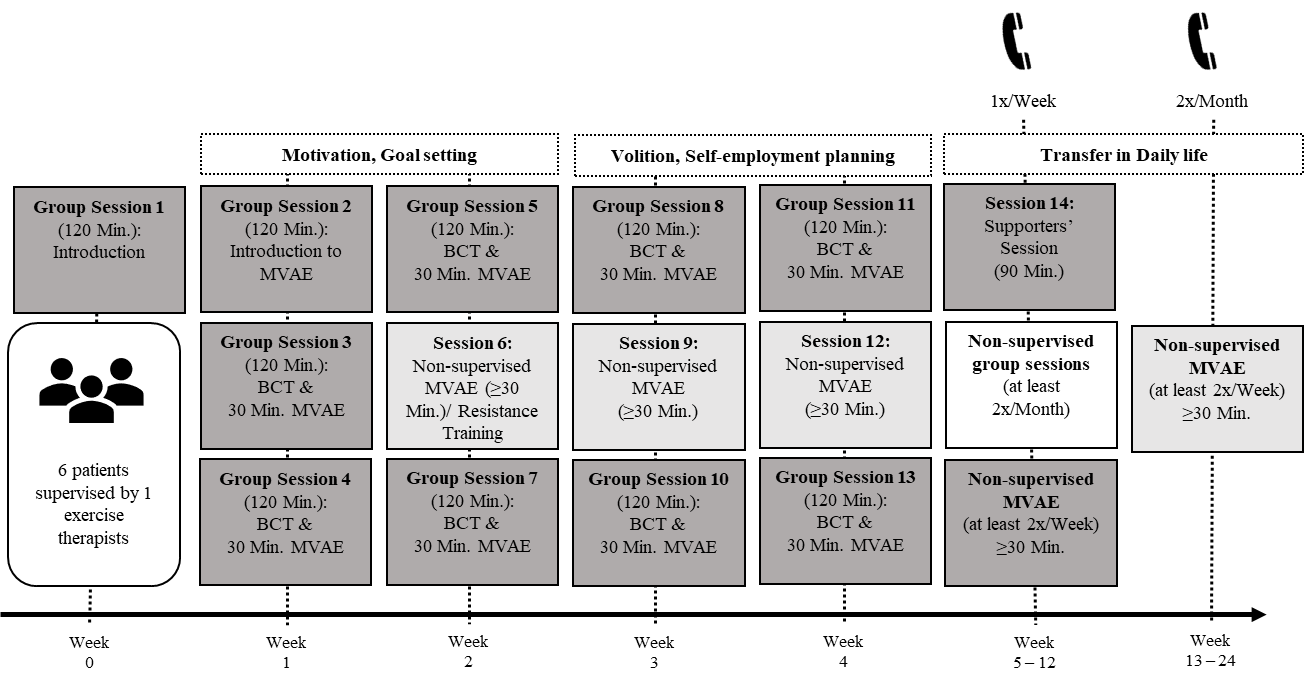
**

Figure 1: The temporal program structure and content overview of ImPuls.

The temporal program structure and content overview of ImPuls. The dark gray boxes illustrate the supervised sessions with group meetings (“Group Session") and moderate to vigorous aerobic exercise (“MVAE”) as well as the supporters’ session in week 5. The group sessions integrate different behavioral change techniques (“BCT”) to enhance motivational and volitional skills with the long-term aim for maintenance aerobic exercise. The medium gray boxes illustrate non-supervised aerobic exercise in which the patients can choose independently any aerobic exercise that best fits to their interests and needs. The light gray box illustrates the non-supervised group sessions from week 5 to 24 in which patients complete the aerobic exercise together but without the therapist. The telephones cartoons represent telephone contacts during the non-supervised time to monitor the long-term maintenance of aerobic exercise. The entire program is supported by the ImPuls smartphone application, developed especially for ImPuls

ImPuls smartphone application (“ImPuls-App”). The ImPuls App will support the participants with options for exercise planning (training plans), exercise guidance (interval training, resistance program, ratings of perceived exertion), self-monitoring of goal achievements (analysis and feedback of FITT criteria and goal achievements), mitigation of barriers and a knowledge base. A web-based application for exercise therapists will support planning and logging individual and group sessions with clients (calendar, attendance, active participation, notes). Furthermore, the participants can share some or all of their data (such as their exercise schedule or their plans for overcoming barriers) with their exercise therapists via the secure channels between the ImPuls App and the web-based platform. This will enable direct feedback of therapists to their clients. Both Apps run on Google Android and on Apple iOS. All data generated in the apps will be protected by encryption on the smartphone. All communication between smartphones (ImPuls App), browsers (Therapist’s App) and the central server will be protected by established encryption protocols, too. All components in table 1 will be documented in or provided by the ImPuls App.

**Table 1.** Overview of behaviour change techniques included in ImPuls

| **Focus** | **Technique** |
| --- | --- |
| Motivational  (mainly weeks 1-2) | Education about positive and negative effects of exercise |
|  | Education about optimal modalities of exercise to experience positive psychological effects |
|  | Selection of a preferred activity and level of intensity  Self-monitoring of exercise  Imagination of goals being reached in the future |
|  | Goal setting |
|  | Self-monitoring of goal achievement |
|  | Reflection about positive experiences/effects with/of exercise  Reflection about self-monitoring of exercise |
| Volitional  (mainly weeks 3-4) | Identification of barriers to exercise |
|  | Techniques to overcome barriers |
|  | Exercise planning through training plans |
|  |  |
| Motivational  and volitional  (weeks 4-24) | Social support (family, friends) through the supporters meeting  Social support (other patients, self-organized group meetings) |
|  | Self-monitoring of goal achievement |
|  | Exercise self-monitoring trough diaries, training plans and analysis of FITT criteria (optimal modality) |

All techniques/approaches will be delivered via the exercise therapists and protocoled, supported and guided by the ImPuls App.

*Item 11b Criteria for discontinuing or modifying allocated interventions for a given trial participant (eg, drug dose change in response to harms, participant request, or improving/worsening disease)*

Participants who miss more than 4 sessions in a row (of w0 to w4; supervised-phase) of the intervention (≥40%, due to any reason) but continue the study assessments are defined as **treatment-dropout**, since they did not receive the intended dose of the intervention. They can still participate in the remaining sessions of the intervention and will be asked to complete all remaining assessments. Participants who miss more than 4 sessions in a row (of w0 to w4; supervised-phase) of the intervention (≥40%, due to any reason) and discontinue the study assessments are defined as **study-dropout**. All participants agree to be asked to specify their reasons for study/treatment-dropout on a voluntary basis (once, after treatment/study-dropout). Reasons for discontinuation will be reported.

If more than 50% of participants discontinue their participation in the intervention group during the first five weeks (w0 to w4) of the delivery, discontinuation of the group due to lack of economic efficiency for the study sites is possible. If only one participant of the intervention group remains, he or she can no longer receive the intervention, since delivery of the intervention to less than two participants no longer qualifies as “group-based intervention”.  All participants, including drop-outs, will be asked to complete all of the following study assessments on a voluntary basis.

*Adherence*

*Item 11c: Strategies to improve adherence to intervention protocols, and any procedures for monitoring adherence (e.g., drug tablet return; laboratory tests).*

**Intervention**

To ensure adequate delivery of the intervention, all exercise therapists were trained in the ImPuls program (see eligibility criteria). All exercise therapists are offered to take part in regular supervisions by a licensed exercise therapist or supervisor in cognitive-behavioral therapy, who are also trained in the ImPuls program by the study PI. All intervention sessions will be video-taped. 10% of the recorded sessions (randomly selected from pre-defined core-sessions) will be evaluated for treatment-fidelity by trained assessors. Every participant will set a 30-minute timer to monitor the duration of the supervised physical activity sessions. During the physical activity, intensity will be monitored through heart-rate monitors (w0-w4) as well as RPE (w0-w24). RPE will be assessed through automatic reminders of the smartphone application after 5, 15 and 25 minutes of physical activity.

The participants’ attendance will be monitored through the smartphone application as well as participation lists. If participants miss a session of the intervention, they will be asked to complete 30 minutes of moderate to vigorous physical activity on their own. They will be sent the material of the patient training session. All participants are asked to schedule and track their physical activity sessions performed during the supervised as well as unsupervised phase through the smartphone application. They will receive automatic reminders to perform the scheduled physical activity. Exercise therapists will be able to access these activity logs and monitor the physical activity after the supervised phase (w0-w4) through phone contacts. If participants fail to meet their goals, exercise therapists will revise the activity goals and measures to overcome obstacles with the participants. Frequency (at least twice a week), intensity (mean intensity of least 64 %) and duration (at least 30 Min each session) during the supervised period (4 weeks), will be assessed via the ImPuls smartphone application.

**Exercise therapists/study therapists**

Study therapists are instructed to call patients in the case of absence or illness. In the case of sickness of study therapists within week 0-4 of the intervention, they can be represented by another trained therapist. In case of absence of more than 2 weeks, study therapists should be replaced be another trained therapist.

Study therapists are not allowed to take leave in week 1-4 of the intervention. In week 5 to 8 therapists should take leave for max. 1 week. Weeks 9 to 24 there are no rules regarding taking leave. In case of leave study therapists have to be represented by another trained exercise therapist.

**Assessments**

To ensure a good completion rate of the online surveys, automatic reminder emails, with the request to complete the questionnaires, are sent to the participants after a fixed number of days: emails were sent to the participants 5 days after receiving the survey invitation for the prep, pre, rando, inter, post and follow-up assessments and 3 days after receiving the invitation for the weekly assessments. In case surveys were still not completed, automatic emails to inform the responsible research assistants at the University of Tuebingen were sent 7, 8 and 10 days after the dispatch of the survey invitation for prep, pre, rando, inter, post and follow-up assessments. At the 7th Day the University of Tuebingen calls the patient, exercise therapist or manager who did not respond. If the 6th Day is a Saturday the University of Tuebingen calls the patient, exercise therapist or manager on the 8th Day.

For the weekly assessments (week one through 12), participants have 4 days to fill out questionnaires, which are sent on Saturday. On Monday: the participants are reminded to fill out the questionnaires via e-mail. If participants do not respond two weeks in a row, the LMU informs the University of Tuebingen about missing response on Tuesday morning. The participants are then contacted per telephone. If participants do not respond for three weeks in a row without dropping out, participants are contacted again. If participants do not respond for four weeks in a row, participants are not contacted via telephone until post1. If participants respond to one weekly questionnaire after a period of non-response the procedure described above will be applied.

For the assessment after randomization (week 0) and the monthly assessments in the unsupervised intervention phase (week 16, week 20, week 24) participants have 7 days to fill out questionnaires, which are sent on Saturday. On Monday the participants are reminded to fill out the questionnaires via e-mail. The LMU informs the University of Tuebingen about missing response on Tuesday and Thursday morning. The participants are then contacted per telephone.

*Concomitant Care*

*Item 11d: Relevant concomitant care and interventions that are permitted or prohibited during the trial.*

ImPuls will be delivered in addition to standard care/treatment as usual (TAU). Any other standard treatment (psychotherapy, pharmacological interventions) covered by German health insurances are permitted.

## Outcomes

*Item 12: Primary, secondary, and other outcomes, including the specific measurement variable (e.g., systolic blood pressure), analysis metric (e.g., change from baseline, final value, time to event), method of aggregation (e.g., median, proportion), and time point for each outcome. Explanation of the clinical relevance of chosen efficacy and harm outcomes is strongly recommended.*

## Primary Outcome

**Global symptom severity.** Global symptom severity will be assessed by the Global Severity Index (GSI) of the German version of the Brief Symptom Inventory [BSI-18] (Derogatis & Fitzpatrick, 2004). The GSI reflects the general mental distress rating on the symptom scales somatization, depression, and anxiety. Each symptom scale consists of 6 items. Thus, 18 items are rated on a 5-point Likert scale (range: 0-4). Higher scores indicate higher distress. Cut-off scores were evaluated separately for men (≥ 10) and women (≥ 13) and have high sensitivity (91.2%) and specificity (92.6%) (Zabora et al., 2001). Among patients with affective disorders, the GSI has demonstrated good internal consistency (α = .89) and construct validity (r = 0.71). Among patients with anxiety disorders the BSI-18 has an internal consistency of Cronbach’s α = .88 and a construct validity of r = 0.67 (Spitzer et al., 2011).

Secondary Outcome**s**

**Major depressive disorder.** The secondary endpoint *depressive symptoms* will be assessed with the PHQ-9 module, assessing symptoms over the last two weeks with nine items, each of them representing one of the DSM-5 (Diagnostic and Statistical Manual of Mental Disorders) criteria for a depressive episode (Kroenke et al., 2001; Spitzer et al., 1999). All items are rated on a 4-point Likert scale (range: 0-3). The sum of all items represents the total score (range: 0-27). Higher scores indicate higher levels of depression. Regarding depressive symptomology, individuals are classified according to the degree of depression severity: absence of depressive disorder (0-4), mild degree of severity (5-10), medium major depression (10-14), severe major depression (15-19) and most severe major depression (20-27). In medical settings the cut-off of ≥10 is used to detect a major depressive disorder (Kroenke et al., 2001; Kroenke et al., 2010). This cut-off was shown to have a sensitivity and specificity of 88 % and 85 %, respectively (Levis et al., 2019). The scale measuring depressive symptoms has an internal consistency of Cronbach’s α = .87 among a representative German sample (Kocalevent et al., 2013; Kroenke et al., 2001).

**Insomnia / Sleep quality**. Nonorganic insomnia will be assessed with the German version of the Insomnia Severity Index [ISI] (Dieck et al., 2018; Morin, 1993). The ISI consists of seven items and assesses the severity of sleep onset difficulties, sleep maintenance difficulties, early morning awakening, satisfaction with current sleep, interference with daytime functioning, noticeability of impairment attributed to sleep problems and degree of distress or concern caused by the sleep problem of the past two weeks. The total score ranges from 0 to 28 (range of component scores: 0-3), with a higher score reflecting greater insomnia severity. The cut-off score of ≥ 11 has shown a high sensitivity (91.4%) and specificity (84.4%) in identifying insomnia. The ISI has shown an internal consistency of Cronbach’s α = .83 among a representative German sample (Dieck et al., 2018).

Sleep Quality will be assessed with the global sleep quality score of the German version of the Pittburgh Sleep Quality Index (PSQI; Buysse et al., 1989). The global sleep quality score is the sum of seven sleep component scores (range of component scores: 0-3): subjective sleep quality, sleep latency, sleep duration, habitual sleep efficiency, sleep disturbances, use of sleeping medications, and daytime dysfunction. The global sleep quality score can vary from 0 to 21 with a cut-off score of 5, identifying clinically raised sleep impairment (Buysse et al., 1989). It has shown a high sensitivity (98.7%) and specificity (84.4%) in identifying insomnia (Backhaus et al., 2002).

#### **Panic disorder and Agoraphobia.** The seven-item Generalized Anxiety Disorder scale [GAD-7] (Lowe et al., 2008; Spitzer et al., 2006) assesses symptom severity of generalized anxiety during the last 2 weeks, however shows good performance as a screening measurement for panic disorder and agoraphobia (Kroenke et al., 2007; Plummer et al., 2016) and will therefor serve as a measure for panic agoraphobia symptoms. Items are rated on a four-point Likert scale (range: 0-3). The sum of all items represents the total score (range: 0-21), with scores of ≥ 5 representing mild, scores of ≥ 10 moderate and scores of ≥ 15 severe anxiety symptom levels, respectively. The cut-off score of ≥ 10 has shown high sensitivity (89%) and specificity (82%) (Spitzer et al., 2006). Among a representative German sample, the GAD-7 has an internal consistency of Cronbach’s α = .85 (Hinz et al., 2017).

Besides the GAD-7, symptoms of panic disorder and agoraphobia symptoms will be assessed with the three-items subscale panic of the 6-items scale anxiety of the BSI-18 (Derogatis & Fitzpatrick, 2004). Current evidence suggests a four-factor structure of the BSI-18 that retains the somatization and depression symptom scales but splits the anxiety symptom scale in two factors: General anxiety and panic (Andreu et al., 2008; von Brachel et al., 2020). The subscale panic of the BSI-18 consists of three items that are rated on a five-point Likert scale (range: 0-4). Among a German outpatient sample that was surveyed five times after the 2^nd^, 6^th^, 10^th^, 18^th^ and 26^th^ therapy session the best fitting model according to Akaike Information Criterion (AIC) was always the model with four factors, compared to one- and three-dimensional models (von Brachel et al., 2020). Among patients with anxiety disorders, the GSI of the symptom scale anxiety has demonstrated good internal consistency (α = .83) and construct validity (r = 0.67) (Spitzer et al., 2011).

**Posttraumatic Stress Disorder**. To assess symptoms of PTSD, the German version of the PTSD Checklist for DSM-5 (PCL-5) (Kruger-Gottschalk et al., 2017) will be used. The questionnaire is a self-report measure that consists of 20 items corresponding to the DSM-5 criteria for PTSD. Participants report their intensity of symptoms over the past four weeks on a five-point Likert scale (0 = not at all to 4 = extremely; total range 0-80). Higher scores indicate higher levels of PTSD. The German Version shows high internal consistency (α = .95), high test-retest reliability (r = .91) and a high construct validity (r = .77). A cut-off of 33 indicates clinically relevant symptomatology.

#### **Health related quality of life.** Health related quality of life will be assessed by the German version of the EQ-5D-5L questionnaire (Herdman et al., 2011; Hinz et al., 2014). It consists of five items concerning the domains mobility, self-care, usual activities, pain or discomfort and anxiety or depression with five answer alternatives each (range: 1-5). The combinations of the answer alternatives can be described with a five-digit number (i.e. the pattern 11111 indicates the optimal health state). Through the EQ-5D-5L questionnaire Quality Adjusted Life Years (QALY) are captured for the economic evaluation. The EQ-5D-5L has an internal consistency of Cronbach’s α = .86 among German chronic heart failure patients. Current data concerning internal consistency in patients with mental disorders exists for the Spanish version of the EQ-5D-5L. Among Spanish patients with major depression the EQ-5D-5L has an internal consistency of Cronbach’s α = .77 (Bilbao et al., 2021).

**Routine data of the health insurances/health care costs**. For the economic evaluation, patients’ routine data collected 6 months before the intervention, during the time of the intervention, and six months after the intervention will be provided by the two participating statutory health insurers (AOK and TK). This data will include patients’ master data, such as gender and age, as well as patient treatment costs. Parameters for treatment costs will comprise costs of inpatient and outpatient care as well as medication, medicals aids or days of incapacity to work. Routine data for each patient will be provided for the time of the intervention as well as one year prior and one year after. The relevant costs are assessed and aggregated as quantities. In addition, cost parameters resulting from the intervention and the implementation will be considered. Subsequently, routine insurance data will be linked to primary data collected.

**Change of health insurance during study participation.** In the case of a change of health insurance company during the study participation, the corresponding participant is still part of the study and continues to complete the assessments. However, routine data of the health insurances/health care costs cannot be provided.

**Exercise behavior/MVAE.** The assessment of self-reported exercise duration and frequency and the assessment of accelerometer -based moderate to vigorous physical activity will serve as two proxies for MVAE. Exercise in minutes per week will be assessed using the self-report Exercise Activity Index of the Physical Activity, Exercise, and Sport Questionnaire (BSA questionnaire; Fuchs et al., 2015). Participants specify type, duration, and frequency of exercise in the last four weeks. Moderate to vigorous physical activity (MVPA) will be assessed via accelerometer-based sensors (Move 4, movisens GmbH). The sensor assesses physical activity of a person based on kinematic data in three dimensions and atmospheric air pressure. This allows to estimate the amount of physical activities of different intensities for a specified time period based on validated algorithms (Anastasopoulou et al., 2012). Patients will wear the sensors for seven consecutive days. Volume of moderate to vigorous physical activity will be indicated in minutes/week and calculated by the daily physical activity of at least moderate intensity (≥ 3 MET)/minutes.

### Assessments to ensure internal validity

***Symptom severity at randomization.*** Symptom severity might change or fluctuate from the diagnostic interview or pre-assessment to the start of the intervention. In order to ensure clinically relevant transdiagnostic and disorder-specific symptomatology with the start of the intervention, scales that assess all primary and secondary outcomes will be presented again between randomization and group start: BSI-18 (Derogatis & Fitzpatrick, 2004), ISI (Dieck et al., 2018; Morin, 1993), PSQI(Buysse et al., 1989), PHQ-9 (Kroenke et al., 2001; Spitzer et al., 1999), GAD-7 (Lowe et al., 2008; Spitzer et al., 2006), PCL-5 (Kruger-Gottschalk et al., 2017).

**(Serious) Adverse Events.** Adverse events (AE) will be assessed at pre, post-1, and follow-up assessment. AEs and Serious Adverse events (SAEs) can be further reported by patients or therapists at a central phone number. AEs and SAEs will be documented and SAEs will be reported to an independent Data Safety and Monitoring Board, which will discuss adjustments to or discontinuation of the entire study**.**

**~~Treatment Fidelity (adherence to protocol of study therapists).~~** ~~Core elements of the manualized ImPuls intervention have been determined a priori. All inhouse sessions (see fig. 3 and table 1) of all therapists at all study sites will be video-taped despite outdoor MVAE. External research assistants will be trained to rate fidelity based on deliverance as intended in the manual. A treatment fidelity score will be determined as a mean of all ratings. 10 % of all video-taped sessions will be randomly selected and analyzed. A fidelity score of ≥ 90 % is assumed~~.

**Dropout and attendance rates (patients).** A drop-out rate less than 30 % and attendance rate ≥ 80 % is assumed. Attendance within the supervised (weeks 0-4; please refer to fig. 3) and partially supervised (weeks 5-24) period will be assessed via the ImPuls smartphone application and attendance lists by the exercise therapists/study site. Participants who miss more than 4 consecutive sessions during the four weeks of the supervised phase (of weeks 0-4) of the intervention (≥40%, due to any reason) but continue the study assessments are defined as treatment-dropout, since they did not receive the intended dose of the intervention. They can still participate in the remaining sessions of the intervention and will be asked to complete all remaining assessments. Participants who miss more than 4 consecutive sessions during the four weeks of the supervised phase (of weeks 0-4) of the intervention (≥40%, due to any reason) and discontinue the study assessments will be defined as study dropout. Participants who will discontinue the treatment as well as the assessments will be defined as study dropout. All participants agree to be asked to specify their reasons for study/treatment-dropout on a voluntary basis (once, after treatment/study dropout). If available, reasons for discontinuation will be reported. If more than 50% of participants will discontinue their participation in the intervention group during the first five weeks (weeks 0-4) of the delivery, discontinuation of the group due to lack of economic efficiency for the study sites is possible. If only one participant of the intervention group remains, he or she can no longer receive the intervention, since delivery of the intervention to less than two participants no longer qualifies as “group-based intervention”.

**MVAE dose within the supervised period (patients).** Frequency (at least twice a week), intensity (mean intensity of least 64 % of maximum heart rate) and duration (at least 30 minutes of MVAE in each session) of exercise during the supervised period (weeks 1-4) will be assessed via the ImPuls smartphone application.

**Expectations, Motivation and Satisfaction.** Validated scales adapted for use in the context of ImPuls will be employed to assess patients’ outcome expectations (Schulte, 2005), motivation (Schulz et al., 1995) and satisfaction with the intervention (Oei & Green, 2008), as well as exercise therapists’ motivation (Steinmayr & Spinath, 2010), and satisfaction with the intervention (Peters et al., 2002). On all scales, mean scores falling within the upper quartile will be judged as indicative of high motivation for and acceptability of treatment, respectively, in patients and exercise therapists.

**Process Evaluation.** ~~In addition to the goals related to investigating efficacy and cost-effectiveness of ImPuls reported in this study protocol, the overall project pursues additional goals focusing on process evaluation and implementation of ImPuls based on the Medical Research Council Guidance in the routine health care system, which will be described in a separate study protocol.~~

## Quantitative Data

Exercise therapists, managers, and patients receive *online questionnaires* via the web-based data management system REDCap (Harris et al., 2019; Harris et al., 2009) at different time points (see Figure 3, Tables 2 (exercise therapists), 3 (managers), 4 (referring healthcare professionals), 5 (patients). All participants receive an individual web-link via E-mail to access the online survey and have 2 weeks to complete it (except weekly assessments during inter phases 1 - 2, where patients have only 1 week to complete it). Reminders are automatically sent out 5 days after receiving the survey invitation (for weekly assessments: 3 days). We primarily used existing and already validated (in German) measures. If these were not available in German, we translated them using established backward translation procedures utilizing native speakers. In order to gain a more profound insight into the processes of the ImPuls intervention, in some measurements of the exercise therapists, managers and physicians, we adapted the items specifically to ImPuls or developed items ourselves. Moreover, recruitment strategies allow for a variety of healthcare professionals (e.g., psychotherapists and primary care physicians) to refer patients to ImPuls. For this purpose, we ask them via online questionnaires about their opinion regarding exercise in combination with behavior change techniques as a new treatment option for patients with mental disorders.

Data of the *ImPuls smartphone application* is collected continuously during the supervised and partially supervised phase (inter 1 – 3; see figure 2 and table 5). It shows the extent to which patients use the ImPuls smartphone application (frequency) in general as well as regarding different application functions including goal setting, barrier management and training plans (patients‘ integration of core components). Repetitive negative thinking and valence of affect are measured with a self-developed self-assessment manikin prior to and after each supervised and unsupervised exercise session over the entire intervention period. We also collect data from the *web-based ImPuls interface* accompanying the ImPuls smartphone application to record the extent to which exercise therapists have used the tool to review patients’ shared information during supervised and partially-supervised phases.

We receive *documentation data* from the exercise therapists and from the project staff. Exercise therapists are required to document whether all scheduled inhouse sessions (supervised phase) and phone calls (partially supervised phase) were offered or completed. As part of the recruitment process, project staff document how patients became aware of the project, how patients are distributed among outpatient rehabilitative and medical care facilities and patient dropouts and their reasons before and during the study. Recruitment strategies include flyers and posters at the offices of the participating outpatient rehabilitative and medical care facilities, primary care physicians, psychotherapists and physiotherapists as well as a direct approach by health insurers involved in the project. We also disseminate information about the ImPuls intervention through self-help groups, daily newspapers, magazines, student mailing lists and social media.

## Qualitative Data

A *guided semi-structured interview* (Reinders, 2015) was developed with respect to aspects of the MRC framework (e.g., acceptability, fidelity/delivery), empirical considerations prior to the intervention and questions that arose over the course of the intervention (e.g., in conversations with exercise therapists). During the interviews, we ask exercise therapists about their experiences with patients in the ImPuls groups they conducted, their opinions about the program content regarding motivational and volitional BCTs and exercise, the perceived applicability of the program (regarding target group, general conditions in the outpatient rehabilitative and medical care facilities, ImPuls smartphone application and their own qualification) and their opinion on a possible long-term implementation of the ImPuls intervention. Possible reports of specific difficulties in implementing the program can provide us with information on why they may have had to deviate from the manual (adherence). Additionally, it can inform us regarding the areas in which they should have received more training. In summary, the interview focuses on facilitators and barriers for the implementation of the ImPuls intervention from exercise therapists’ perspectives. Interviews are conducted face to face by researchers of the process evaluation team with 20 exercise therapists, who all conducted at least one ImPuls group. The interviews have an estimated average duration of 50 minutes.

A *focus group interview* was developed analogous to the procedure mentioned above. It is supposed to provide an in-depth insight into managers’ perspectives concerning the perceived barriers and facilitators regarding the feasibility of the ImPuls intervention in the outpatient setting and its possible long-term implementation in the future. The focus group interview is conducted with 10 managers and is estimated to last 120 minutes.

Face to face interviews as well as the focus group interview are conducted once there are no further ImPuls groups in the respective outpatient rehabilitative and medical care facility. All interviews will be audio-recorded. Subsequently, the audio-records are saved on a secured network drive of the University of Tübingen and transcribed verbatim by research assistants. All mentions of personal data are masked during the transcription.

To assess *fidelity*, we record all 10 inhouse sessions of each ImPuls group conducted by the exercise therapists (except outdoor running activity) on video. We will then randomly select one video for each group out of the eight core sessions to evaluate. This corresponds to 12,5% of all core sessions and 10% of all recorded sessions. Randomization is done by an independent person who creates a randomization list using the software R version 4.1.2 (R Core, 2021). We will develop separate rating forms for each ImPuls session. Research assistants of the evaluation team will rate the sessions with regard to adherence to the treatment manual and quality of delivery. Raters will be trained in understanding the ImPuls manual and central features (setting S.M.A.R.T. goals, implement coping plans, execution of 30 minutes of exercise, discussion about perceived exertion, planning of preferred individual exercise training plans). Adherence to the treatment manual is assessed by rating with “yes” (presence) or “no” (absence) as to whether pre-defined core elements of the ImPuls intervention are delivered. An overall inter-rater reliability score is calculated. Adherence is calculated according to the overall amount of “yes”/”no” answers of these items. Subsequently, the percentage of all items answered with “yes” is calculated in order to determine the final adherence percentage score. Quality of delivery is rated once per session on a 4-point Likert scale ([1] totally agree – [4] totally disagree) using four items (exercise therapist listens actively, allows breaks and periods of reflection to take place [tolerates silence], includes all participants, takes statements of participants seriously). A sum score is calculated.

## Participant Timeline

*Item 13: Time schedule of enrolment, interventions (including any run-ins and washouts), assessments, and visits for participants. A schematic diagram is highly recommended.*

**Figure 2:** Patient Flow of the pragmatic randomized controlled trial in accordance with CONSORT

Process evaluation assessment


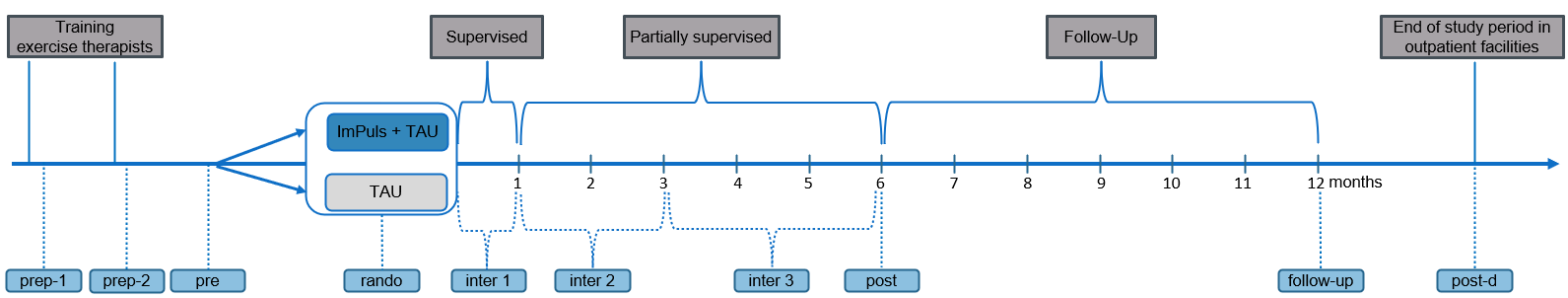


**Figure 3:** Design of the research project including all measurement time points of the process evaluation.

Note (Figure2): follow-up = 12 months after intervention start; Inter-1 = supervised ImPuls phase (week 1-4); inter-2 = partially supervised ImPuls phase 1 (week 5-12); inter-3 = partially supervised ImPuls phase 2 (week 13-24); post = end of the intervention (week 24); post-d = end of study period in the outpatient facility; pre = prior to intervention start in the outpatient facility; prep-1 = following the first training; prep-2 = following the second training; rando = after randomization, prior to intervention start in the outpatient facility.
TAU (=Treatment as usual) is assessed only at pre, post, and follow-up. Assessments during inter 1 (week 1 – 4), inter 2 (week 5 – 12) and inter 3 (week 13 – 24) concern only exercise therapists and patients. Assessment frequency within inter-assessments punctually differs between participants (see tables 1 – 4).

**Table 2:** Measurements for exercise therapists at each time point (following SPIRIT template).

| ASSESSMENT EXERCISE THERAPISTS | TIMEPOINT | | | | | | | | |
| --- | --- | --- | --- | --- | --- | --- | --- | --- | --- |
|  | prep-1 | prep-2 | pre | inter-1 | inter-2 | inter-3 | post | follow-up | post-d |
| Implementation |  | | | | | | | | |
| Training – satisfaction (Göhner et al., 2018) [modified] | x | x |  |  |  |  |  |  |  |
| Training – perceived skill acquisition / self-efficacy (ImPuls intervention) (Resnick & Jenkins, 2000; Rigotti et al., 2008; Rodgers et al., 2008) [adapted to Impuls] | x | x |  |  |  |  |  |  |  |
| Supervision - participation rate |  |  |  | x |  |  | x |  | x |
| Supervision – satisfaction (Göhner et al., 2018) [modified] |  |  |  | x |  |  | x |  | x |
| App - user frequency (App) |  |  |  | x | x | x |  |  |  |
| Adherence (video) |  |  |  | x |  |  |  |  |  |
| Quality of delivery (video) |  |  |  | x |  |  |  |  |  |
| Dosage - amount sessions delivered (documentation) |  |  |  | x | x | x |  |  |  |
| Mechanisms of impact |  | | | | | | | | |
| Global self-efficacy ImPuls (Rigotti et al., 2008) [adapted to Impuls] |  |  | x |  |  |  |  |  | x |
| Attitudes towards mental disorders (OMS-HC; Modgill et al., 2014) [translated + modified] |  |  | x |  |  |  |  |  | x |
| Attitudes towards manualized interventions (EBPAS-36D; Szota et al., 2021) |  |  | x |  |  |  |  |  |  |
| Motivation (SESSW; Steinmayr & Spinath, 2010) [modified]; (self-developed items) |  |  | x | x |  |  |  |  | x |
| Expectation of program success (PATHEV; Schulte, 2005) |  |  | x |  |  |  |  |  |  |
| Program acceptance/satisfaction (B&F-A; Harmsen et al., 2005) [translated + modified] |  |  | x |  |  |  |  |  | x |
| Perceived barriers (B&F-A; Harmsen et al., 2005) [translated + modified] |  |  |  | x | x |  | x |  |  |
| Satisfaction with the ImPuls group (Göhner et al., 2018, WAI-SR; Wilmers et al., 2008) [modified] |  |  |  | x |  |  | x |  |  |
| Coping strategies (DPCCQ; Orlinsky et al., 1999) [modified] |  |  |  | x |  |  | x |  |  |
| App-usability (SUS; Liang et al., 2018) [translated + modified] |  |  |  | x | x |  | x |  |  |
| App-functionality (MARS-G;Messner et al., 2020) |  |  |  |  |  |  | x |  |  |
| App-viability (self-developed) |  |  |  |  |  |  | x |  |  |
| App-satisfaction (MARS-G;Messner et al., 2020) |  |  |  | x | x |  | x |  |  |
| Context |  | | | | | | | | |
| Demographics | x |  |  |  |  |  |  |  |  |
| Qualification (self-developed) | x |  |  |  |  |  |  |  |  |
| Therapeutic experience (regarding exercise in group setting /with patients with mental disorders) (self-developed) | x |  |  |  |  |  |  |  |  |
| *Note:* follow-up = once after 12 months; Inter-1 = once at the end of the supervised ImPuls phase (week 1-4); inter-2 = once at the end of the partially supervised ImPuls phase 1 (week 5-12); inter-3 = once at the end of the partially supervised ImPuls phase 2 (week 13-24); post = after completion of the intervention (week 24-26, supervised and partially supervised) ImPuls phases; post-d = end of study period in the outpatient facility; pre = prior to intervention start in the outpatient facility; prep-1 = following the first training; prep-2 = following the second training; rando = after randomization, prior to intervention start in the outpatient facility.  *Abbreviations:* B & F – A= Barriers and facilitators assessment instrument; DPCCQ = Development of Psychotherapist Common Core questionnaire; EBPAS -36D = Evidence based practice attitude scale (German version); FPTM-40= Therapy motivation questionnaire; MARS-G = Mobile App Rating Scale (German version); OMS-HC = Opening minds scale for Health Care Providers; PATHEV = Measurement of therapy expectation and therapy evaluation of patients; SESSW = Scale for recording subjective school values; SUS = System usability scale; WAI-SR = Working Alliance Inventory – short revised (German version). | | | | | | | | | |

**Table 3:** Measurements for managers at each time point (following SPIRIT template)

| ASSESSMENT MANAGERS | TIMEPOINT | | | | | | | | |
| --- | --- | --- | --- | --- | --- | --- | --- | --- | --- |
|  | prep-1 | prep-2 | pre | inter-1 | inter-2 | inter-3 | post | follow-up | post-d |
| Context |  | | | | | | | | |
| Qualification (self-developed) |  |  | x |  |  |  |  |  |  |
| Structural characteristics of outpatient facilities (Deutsche Rentenversicherung Bund (DRV), 2014; Geidl et al., 2018; GKV Spitzenverband) [modified] |  |  | x |  |  |  |  |  |  |
| Motivation (self-developed) |  |  | x |  |  |  | x |  | x |
| Satisfaction (Göhner et al., 2018; Johnson & Caldwell, 2011) [modified] |  |  |  |  |  |  | x |  | x |
| Perceived barriers (B&F-A; Harmsen et al., 2005) [translated + modified] |  |  | x |  |  |  | x |  | x |
| *Note:* follow-up = once after 12 months; Inter-1 = once at the end of the supervised ImPuls phase (week 1-4); inter-2 = once at the end of the partially supervised ImPuls phase 1 (week 5-12); inter-3 = once at the end of the partially supervised ImPuls phase 2 (week 13-24); Post = after completion of three ImPuls intervention groups in the outpatient facility; post-d = end of study period in the outpatient facility; pre = prior to intervention start in the outpatient facility; prep-1 = following the first training; prep-2 = following the second training; rando = after randomization, prior to intervention start in the outpatient facility.  *Abbreviations:* B & F – A= Barriers and facilitators assessment instrument | | | | | | | | | |

**Table 4:** Measurements for referring health care professionals at each time point (following SPIRIT template)

| ASSESSMENT REFERRERS | TIMEPOINT | | | | | | | | |
| --- | --- | --- | --- | --- | --- | --- | --- | --- | --- |
|  | prep-1 | prep-2 | pre | inter-1 | inter-2 | inter-3 | post | follow-up | post-d |
| Implementation |  | | | | | | | | |
| Professional background (self-developed) |  |  |  |  |  |  |  |  | x |
| Opinion on the new treatment option (Kien et al., 2021); (self-developed items) |  |  |  |  |  |  |  |  | x |
| Physical activity level (EHIS-PAQ; Finger et al., 2015) [modified] |  |  |  |  |  |  |  |  | x |
| *Note:* follow-up = once after 12 months; Inter-1 = once at the end of the supervised ImPuls phase (week 1-4); inter-2 = once at the end of the partially supervised ImPuls phase 1 (week 5-12); inter-3 = once at the end of the partially supervised ImPuls phase 2 (week 13-24); post = after completion of the intervention (week 24-26, supervised and partially supervised) ImPuls phases post-d = after end of study period in all outpatient facilities; pre = prior to intervention start in the outpatient facility; prep-1 = following the first training; prep-2 = following the second training; rando = after randomization, prior to intervention start in the outpatient facility.  *Abbreviations:* EHIS-PAQ= European Health Interview Survey - Physical Activity Questionnaire | | | | | | | | | |

**Table 5:** Measurements for patients at each time point (following SPIRIT template)

| ASSESSMENT PATIENTS | TIMEPOINT | | | | | | | | | |
| --- | --- | --- | --- | --- | --- | --- | --- | --- | --- | --- |
|  | prep-1 | prep-2 | pre | rando | inter-1 | inter-2 | inter-3 | post | follow-up | post-d |
| Implementation |  |  |  |  |  |  |  |  |  |  |
| demographics |  |  | x |  |  |  |  |  |  |  |
| Mechanisms of impact |  | | | | | | | | | |
| *Responses and interactions^1^* |  |  |  |  |  |  |  |  |  |  |
| Motivation (FPTM-40; Schulz et al., 1995) [modified] |  |  |  | x |  |  |  |  |  |  |
| Expectation of program success (PATHEV; Schulte, 2005) [modified] |  |  |  | x | x | x | x |  |  |  |
| Satisfaction with the program (SSTS-R; Oei & Green, 2008) [translated + adapted to Impuls] |  |  |  |  | x | x | x |  |  |  |
| Therapeutic alliance (WAI-SR; Wilmers et al., 2008) |  |  |  |  | x | x | x |  |  |  |
| App |  |  |  |  |  |  |  |  |  |  |
| User frequency (App) |  |  |  |  |  |  |  |  |  |  |
| Usability (SUS; Liang et al., 2018) [translated + modified] |  |  |  |  | x | x | x |  |  |  |
| Functionality (MARS-G; Messner et al., 2020) |  |  |  |  |  |  | x |  |  |  |
| Viability (self-developed) |  |  |  |  |  |  | x |  |  |  |
| Satisfaction (MARS-G; Messner et al., 2020) |  |  |  |  | x | x | x |  |  |  |
| Attendance rate (documentation) |  |  |  |  | x |  |  |  |  |  |
| Treatment dropouts (documentation) |  |  |  |  | x | x | x |  |  |  |
| *Mechanisms of change* |  |  |  |  |  |  |  |  |  |  |
| Emotional intelligence (TEIQue; Petrides & Furnham, 2006) |  |  | x |  |  |  |  | x | x |  |
| Emotion regulation (DERS; Gratz & Roemer, 2004) |  |  | x |  |  |  |  | x | x |  |
| Barrier management (Sniehotta et al., 2005) |  |  | x |  |  |  |  | x | x |  |
| Perceived stress (PSS; Schneider et al., 2020) |  |  | x |  |  |  |  | x | x |  |
| Physical activity-related health competence (PAHCO; Sudeck & Pfeifer, 2016) |  |  | x |  |  |  |  | x | x |  |
| Repetitive negative thinking (PTQ; Ehring et al., 2011) ^2^ |  |  | x |  | x | x | x | x | x |  |
| Affect (state/trait) (PANAS; Breyer & Bluemke, 2016)^1, 2^ |  |  | x |  | x | x | x | x | x |  |
| Affect/repetitive negative thinking during the program (App)^1, 3^ (self-developed) |  |  |  |  | x | x | x |  |  |  |
| Goal attainment during the program (App)^1, 3^ (visual analogue scale) |  |  |  |  | x | x | x |  |  |  |
| Barrier management during the program (App)^1, 3^ (visual analogue scale) |  |  |  |  | x | x | x |  |  |  |
| Frequency, intensity, time/duration and type of physical activity (FITT criteria, App)^1, 3^ |  |  |  |  | x | x | x |  |  |  |
| *Note*: follow-up = once after 12 months; Inter-1 = once at the end of the supervised ImPuls phase (week 1-4); inter-2 = once at the end of the partially supervised ImPuls phase 1 (week 5-12); inter-3 = once at the end of the partially supervised ImPuls phase 2 (week 13-24); post = after 6 months; post-d = end of study period in the outpatient facility; pre = before randomization, prior to intervention start in the outpatient facility; prep-1 = following the first training; prep-2 = following the second training; rando = after randomization, prior to intervention start in the outpatient facility ^1^ intervention group only.  ^2^ weekly assessments (inter 1 = 4 times [week 1, 2, 3 and 4]); inter 2 = 8 times [week 5, 6, 7, 8, 9, 10, 11 and 12]) / monthly assessment (inter 3 = 3 times [week 16, 20 and 24]).  ^3^ depending on patient’s usage of the smartphone application, data is collected continuously throughout the phases.  *Abbreviations:* DERS = Difficulties in Emotion Regulation Scale; FITT = Frequency, Intensity, Time/duration and Type of physical activity; FPTM-40= Therapy motivation questionnaire; MARS-G = Mobile App Rating Scale (German version); PAHCO = Physical Activity-related Health Competence questionnaire; PANAS = Positive and Negative Affect Schedule; PATHEV = Measurement of therapy expectation and therapy evaluation of patients; PSS = Perceived Stress Scale; PTQ = Perseverative Thinking Questionnaire; SSTS-R = Satisfaction with therapy and therapist scale (revised); SUS = System Usability Scale; TEIQue = Trait Emotional Intelligence Questionnaire ; WAI-SR = Working Alliance Inventory – short revised (German version). | | | | | | | | | | |

## Sample Size

*Item 14: Estimated number of participants needed to achieve study objectives and how it was determined, including clinical and statistical assumptions supporting any sample size calculations.*

The sample size was determined a priori using power analysis (G*Power, version 3.1.9.2) (Faul et al., 2009; Faul et al., 2007). Power analysis was conservatively based on the lowest symptom-related post-treatment effect of exercise (vs. TAU/waiting list) on all included clinical disorders, namely the effect size of d = -0.348 (g = - 0.347) for symptoms of post-traumatic stress disorder (Rosenbaum et al., 2015). A two-sided *t*-test, alpha level of 0.05, a test power of 80%, an equal cell population, and a dropout rate of 30% were assumed. This calculation resulted in N = 375, which is conservative enough to detect the lowest expected treatment effect at the post-treatment phase. However, we regarded this sample size as the minimum and targeted a total sample of N = 600, in order to have enough statistical power for further analysis performed in the course if the process evaluation (see Statistical Methods – Items 20 a, b and c). ~~that will be predefined and published in a separate study protocol regarding the evaluation of the entire implementation process based on the Medical Research Council framework.~~

## Recruitment

*Item 15: Strategies for achieving adequate participant enrolment to reach target sample size.*

Patients will be recruited mainly via inpatient psychiatric departments, family practices, general practitioners and psychiatric and psychotherapeutic outpatient units. The project will be conducted in collaboration with two health insurances, the AOK Baden-Württemberg (AOK) and the Techniker Krankenkasse (TK), who will support the recruitment with a targeted approach of general practitioners, psychiatrists and psychotherapists. All hospitals, clinics and practices will receive information material, such as flyers and posters, to inform their patients about the project. In addition, AOK and TK will publish articles in media distributed for their members during the course of the project. The TK further will inform eligible patients directly via phone calls. Recruitment will be additionally performed via social media posts (Instagram and Facebook), newsletters of professional associations, email distribution lists of universities, local influencers and regional newspapers and television.

Interested patients will first attend a preliminary telephone screening of eligibility criteria and will receive general information about the project (see also fig. 2). Patients will be screened for somatic contraindications for exercise via the Physical Activity Readiness Questionnaire (PARQ) (Thomas et al., 1992) and will be informed that they have to provide a physician referral for ImPuls before pre-assessment. In case of any suspicion of somatic contraindications, that might oppose participation (e.g., heart diseases or orthopedic problems), patients will be asked to provide an additional medical consult from their general practitioner or a medical specialist. Eligible participants will be invited for a first inhouse meeting taking place in a study site close to their residence. Within the meeting, they will provide informed consent for study participation, receive information about the study site and will be screened initially for symptomatology related to the exclusion criteria to prepare for the structural diagnostic interview. The telephone screening and initial interview will be performed by trained research assistants. Following this first inhouse meeting, psychologists with a M.Sc. degree undergoing a training in cognitive-behavior therapy, who will be trained by an external expert for structured clinical interviews, will conduct the structured clinical interview for DSM-5 (SKID-5-CV) (Beesdo-Baum et al., 2019) to confirm eligibility.

Once six patients at the same site will be found to be eligible for participation, they will receive online questionnaires via the web-based data management system REDCap (Harris et al., 2019; Harris et al., 2009) and a accelerometer-based Physical Activity sensors (MOVE 4; movisens GmbH) which will be worn for seven consecutive days (pre-assessment). Online questionnaires and the assessment of physical activity will be carried out within a period of 14 days. On Day 15, the six patients will be randomized as a group to either the intervention or control condition. In case of an assignment into the intervention condition, study sites will have to start the intervention within 14 days. Before the start of the intervention, global symptom as well as disorder-specific symptom severity will be assessed again to check whether participants meet the cut-off criteria for a mental disorder. In each study site, 60 patients are planned to be recruited and randomized, resulting in 10 allocations per site. The intervention group will complete the exercise intervention in addition to TAU, while the control group will receive TAU within the real-world outpatient mental health care setting in Germany. The TAU condition is intended to represent the typical treatment patients receive in the German outpatient health care system. The procedure of the pre-assessment phase will be repeated six months (Post) and 12 months (Follow-up) after randomization. After the completion of all assessments, patients of the control group will receive 450€ as reimbursement for their time.

## Allocation

### Sequence Generation

*Item 16a: Method of generating the allocation sequence (e.g., computer-generated random numbers), and list of any factors for stratification. To reduce predictability of a random sequence, details of any planned restriction (e.g., blocking) should be provided in a separate document that is unavailable to those who enrol participants or assign interventions.*

### Concealment Mechanism

*Item 16b: Mechanism of implementing the allocation sequence (e.g., central telephone; sequentially numbered, opaque, sealed envelopes), describing any steps to conceal the sequence until interventions are assigned.*

### Implementation

*Item 16c: Who will generate the allocation sequence, who will enrol participants, and who will assign participants to interventions.*

The randomization sequence is generated independently of the study coordinator and the research team responsible for data collection and management. The sequence is generated using a varying-size permuted block design, stratified by study site. This procedure ensures an appropriate balance in the number of treatment and control groups per study site. Randomization codes are generated digitally and concealed on a secure system. The group-allocation sequence is concealed from the research team responsible for data collection and management until the planned unblinding.

## Blinding (Masking)

*Item 17a: Who will be blinded after assignment to interventions (e.g., trial participants, care providers, outcome assessors, data analysts), and how.*

*Item 17b: If blinded, circumstances under which unblinding is permissible, and procedure for revealing a participant’s allocated intervention during the trial.*

Patients are not blind to allocation. No assessors need to be blinded since primary and secondary outcomes are not based on clinician/diagnostician ratings. The sponsor and his research team responsible for data collection and management and any personal in contact with patients is blinded regarding the randomization procedure. The data analyst is blinded regarding the allocation. He receives the final dataset, which is masked for the treatment condition (the “condition” variable only informs Condition A or B but no real labels of the treatment and control conditions). An unblinded data manager handles the raw data when exporting the data from REDCap.

## Data Collection Methods

*Item 18a: Plans for assessment and collection of outcome, baseline, and other trial data, including any related processes to promote data quality (e.g., duplicate measurements, training of assessors) and a description of study instruments (e.g., questionnaires, laboratory tests) along with their reliability and validity, if known. Reference to where data collection forms can be found, if not in the protocol.*

Please refer to *item 11 d* **“Outcomes”**

*Item 18b: Plans to promote participant retention and complete follow-up, including list of any outcome data to be collected for participants who discontinue or deviate from intervention protocols.*

In accordance with the Evaluation Concept and Record of Processing Activities, all data for evaluation runs through REDCap on a secured LMU internal server providing a stable and traceable coding, storage and security structure as well as data quality measures such as automatic survey reminders, alerts to researchers for telephone reminders to participants, and data type and range checks where appropriate. Missing values on surveys have to be confirmed by participants to be intentional before being able to proceed to the next questionnaire.

The process evaluation data collected via app are stored pseudonymously on a server of the Central Office of the University of Tuebingen (Tue-CO) and transmitted pseudonymously to LMU-Psy. Likewise, videos of the group sessions will be recorded by Tue-MA as part of the process evaluation. These videos will be stored on a hard-drive at the Tü-CO and transferred to the LMU-Psy via RedCap. The routine data of the health insurances (AOK/TK) include outpatient, inpatient, as well as medicines, remedies and aids six months prior and up until six months after completion of the intervention period and are transmitted to the trust center (TUM-TC) on the basis of a pseudonym assigned by the Tue-CO, which is not known to the patients, and from there double pseudonymized to the TU Munich (TUM-HSM). The data stored at LMU-Psy (primary and secondary endpoints, data on progression diagnostics and process evaluation) are transmitted to TUM-TC via a secure connection. From there, they are transmitted to TUM-HSM with the routine data and will be double pseudonymized.

## Data Management

*Item 19: Plans for data entry, coding, security, and storage, including any related processes to promote data quality (e.g., double data entry; range checks for data values). Reference to where details of data management procedures can be found, if not in the protocol.*

**Data Entry.** Participant records are created in REDCap by Tue-MAand include pseudonym, e-mail address, study center, metadata and assessment dates. Patient records further include diagnostics and exercise therapist records include dropout-dates as well as dropout-reasons.

The data that participants enter in the smartphone application during their participation in the trial are securely stored on their phones. The data are also transmitted to a central server located at University of Tuebingen. Participants are able to share some or all of their data (that is directly related to their participation such as exercise schedules or plans for goal achievement) with their exercise therapist. They can access a summary of the participant’s data via a browser-based application in order to plan their group session or provide feedback and guidance to the individuals.

Randomization by LMU personnel is automatically triggered 2 weeks after Pre-assessment. Participants enter all questionnaire data electronically via REDCap from a date-triggered REDCap invitation link. AE and Dropout-data are entered by staff of the sponsor after notification from the therapist or the participant.

**Data Quality**. Data quality is ensured through several mechanisms: Validity, data and range rules are installed for data entry in REDCap wherever possible. All manual modification and changes to the database will be documented in electronic logs in REDCap and only e-mail addresses will be deleted from logs after data collection. Participants have to confirm it to be an intentional choice if they leave items unanswered on questionnaires. Automatic date-triggered reminders are sent to participants for questionnaires. Tue-MA receive date-triggered alerts for phone-reminders if questionnaires are still missing. Additionally, regular checks by the evaluating center help detect and prevent data discrepancies. The evaluating center will regularly send reports about missing data to the University of Tuebingen.

The trust center ensures the data quality of the routine data used for the economic evaluation by controlling for completeness of the dataset and plausibility of selected variables by calculating means and distributions.

The data transmitted from the smartphone of the participants to the server are stored in a database. New records are appended to the database and do not overwrite previous records. Faulty records can thus be interpolated with the previous and the next one. Established technical measures prevent corruption of data during transmission.

**Data Security.** A data protection concept was developed together with all partners, which was approved by the data protection officers of the University of Tuebingen, the LMU, TU and from the 2 health insurances AOK and TK. The concept can be provided upon request in German language. Violations of data privacy will be documented and reported to an external data protection officer.

The participant’s data are automatically deleted upon deletion of the study smartphone application. Transport to the server is protected by established encryption protocols. Access to the server site is protected via technical and organizational measures of the University of Tuebingen. Access to the data is only granted to specific personell responsible for technical maintenance.

All study personnel are restricted to their user rights to the necessary tasks only, i.e. LMU quality control team cannot access participants’ e-mail address while Tuebingen personnel don’t have access to participants’ questionnaire data. REDCap communication channels are encrypted, and all REDCap Data is stored on a secured internal server with logging and regular backups, which is maintained by the faculty’s IT. REDCap itself is regularly updated.

Original paper versions are safely stored in Tuebingen for 10 years after the completion of the study.

Data to and from the trust center will be transmitted via a secure online connection. Only pseudonymous data is transmitted. For data quality checks the data will be stored and analyzed on an external server without any network connections. Thereafter, data will be locked on an external hard drive within a Veracrypt container and kept within a steel safe. Data is permanently erased one year after the completion of the study.

## Statistical Methods

*Item 20a: Statistical methods for analyzing primary and secondary outcomes. Reference to where other details of the statistical analysis plan can be found, if not in the protocol.*

*Item 20b: Methods for any additional analyses (e.g., subgroup and adjusted analyses).*

*Item 20c: Definition of analysis population relating to protocol non-adherence (e.g., as randomised analysis), and any statistical methods to handle missing data (e.g., multiple imputation).*

To test Hypotheses 1, 2, 3a and 4 we will use multilevel modeling to establish the treatment effects on the primary outcome (global symptom severity) and the secondary outcomes (major depressive disorders, insomnia, panic disorder, agoraphobia, PTSD, QALYs, exercise). In these analyses, each outcome will be predicted by the group (ImPuls + TAU vs. TAU), time (pre vs. post vs. follow-up), and their interaction. Given that the randomization is stratified by study site, we will account for the effects of study site in all analyses. All analyses will be performed on intention-to-treatment basis, using maximum likelihood estimations.

To test Hypothesis 3b, mediation analyses will be performed to test the indirect effects of the treatment on the primary outcome that are mediated by the changes in the putative mediators (self-reported exercise in minutes per week, accelerometry-based moderate to vigorous physical activity (MVPA); from pre to post, pre to follow-up and post to follow-up). We will compute standardized change scores for the outcome and mediators (Lee et al., 2019). In a path model (specified in the framework of structural equation modeling), the group variable predicts the changes in the mediators, and these mediating factors further predict the change score in the outcome. The indirect effects are defined by the products of the group-mediator and mediator-outcome effects. As exploratory analyses, we will also examine other forms of mediations proposed by Goldsmith et al. (Goldsmith et al., 2018), encompassing cross-lagged effects and latent change score models. As recommended by Usami et al.(Usami et al., 2019), we will first test whether these models fit the data well (and which model fits the data best), and then investigate the indirect effects of the treatment on the outcome.

To further analyze clinical significant changes (additional analysis for Hypothesis 4 and 5), the Reliable Change Index (RCI) (Balkin & Lenz, 2021; Jacobson & Truax, 1992; Zahra, 2010) will be computed for each individual between pre-, post and follow-up assessments. Jacobson and Truax suggest combining the RCI with an estimation of a cut-off point between a functional (nonpatient) and dysfunctional (patient) population, describing a clinically significant change (Jacobson et al., 1984; Jacobson & Truax, 1992). We will check if individual scores are more than 2 SD away from the mean of the complete sample. Each individual will be classified into one of four categories: recovered (individual has passed cut-off point of clinically significant change and the RCI is greater than 1.96), improved (individual has not passed cut-off point but the RCI is greater than 1.96), unchanged (individual has not passed neither cut-off point nor is the RCI greater than 1.96), or deteriorated (the individual’s RCI is greater than -1.96). Non-parametric generalized mixed models will be conducted to analyze differences between intervention and control group.

Concerning process evaluation:

Empowerment of exercise therapists (research question 1a) will be analyzed descriptively (mean, standard deviation) with respect to their self-reports on the modified training evaluation scale (Göhner et al., 2018), the (occupational-) self-efficacy scale (Rigotti et al., 2008) and concerning the frequency of supervision.

We descriptively present the adherence score (mean, standard deviation) as well as the corresponding percentage to check whether the desired high level of adherence (>90%) in the context of the efficacy trial is achieved (research question 1b).

We descriptively present the amount of all patients acquired by each strategy as well as the amount of all patients included in the study by each strategy. We then check which recruitment strategy has the highest inclusion rate and present the corresponding percentage (research question 1c).

Referring healthcare professionals’ opinion on the new treatment option is presented descriptively (mean, standard deviation) (research question 1d).

To provide information about the dose delivered (research question 1e), we present documentation data of exercise therapists and the web-based ImPuls interface descriptively.

To assess barriers and facilitators (research question 2a), we use quantitative data from questionnaires (barriers and facilitators assessment instrument (B&F-A; Harmsen et al., 2005), satisfaction scale (Göhner et al., 2018; Johnson & Caldwell, 2011), as well as basic recommendations for outpatient facilities in Germany (Deutsche Rentenversicherung Bund (DRV), 2014; Geidl et al., 2018; GKV Spitzenverband)). Results are presented descriptively (mean, standard deviation) to provide an overview over contextual characteristics. This data is further complemented with qualitative data from the interviews.

We evaluate the interviews in a deductive-inductive process following the steps of a content-structuring qualitative content analysis (Kuckartz, 2016). First, two researchers will elaborate a preliminary coding frame for coding based on the interview guideline. Subsequently, 15% of all interviews are independently coded by those researchers. Inter-coder-reliability analysis is then performed to ensure that the elaborated coding frame is applicable. Potential discrepancies are discussed to refine the coding frame in an iterative process. This process is continued until both researchers agree that the categories are distinct and no new categories need to be added to the coding frame. Afterwards, the remaining interviews are coded. Coding and analysis are done by using the software MAXQDA 2022 (VERBI, 2021). In a final step, the statements from all interviews are summarized category by category and used to supplement the quantitative data.

We will conduct mediation and moderation analyses as well as subgroup analyses to gain deeper insight into the impact of the program (mechanisms of change). We check whether exercise therapists’ attitude towards mental disorders (Opening minds scale for health-care providers (OMS-HC; Modgill et al., 2014)), evidence-based practice (evidence-based practice attitude scale – German version (EBPAS-36D; Szota et al., 2021)) and the program ( measurement of therapy expectation and therapy evaluation of patients (PATHEV; Schulte, 2005), B&F-A) affects treatment effects (research question 3a). In addition, patients’ application data as well as changes in respective individual behavioral determinants (e.g., action and coping plans; Physical activity-related health competencies (PAHCO; Sudeck & Pfeifer, 2016)) will be used to determine the extent to which core components of the intervention have been used and how this affects treatment effects (research question 3b). Further analysis is done to determine the extent to which motivational/volitional core components of the ImPuls intervention (application data [barrier management, goal-setting], documentation data [phone contacts]) as well as changes in respective individual behavioral determinants (e.g., action and coping plans; [PAHCO]) affect patients’ exercise adherence (research question 3c). Finally, we want to explore whether psychological processes such as emotional intelligence (Trait emotional intelligence questionnaire (TEIQue; Petrides & Furnham, 2006)), emotional regulation (difficulties in emotion regulation scale (DERS; Gratz & Roemer, 2004)), repetitive negative thinking (perseverative thinking questionnaire (PTQ; Ehring et al., 2011)) or perceived stress (perceived stress scale (PSS; Schneider et al., 2020)) mediate the treatment effect on global symptom severity (research question 3d).

Missing data will be handled by multiple imputations.

## Data Monitoring

*Item 21a: Composition of data monitoring committee (DMC); summary of its role and reporting structure; statement of whether it is independent from the sponsor and competing interests; and reference to where further details about its charter can be found, if not in the protocol. Alternatively, an explanation of why a DMC is not needed.*

The Data Safety and Monitoring Board is composed of independent researchers who are not associated with the project but who nevertheless have expertise in clinical or medical research. All members of the Data Safety and Monitoring Board are listed in the table below.

| Name | Institution / Position | Adress |
| --- | --- | --- |
| Prof. Dr. Jürgen Hoyer | Dresden University of Technology  Institute of Clinical Psychology and Psychotherapie; Chair of Behavioural Psychotherapie | Hohe Straße 53  01187 Dresden |
| Apl. Prof. Dr. Ferdinand Keller | University Hospital Ulm  Senior psychologist of the child and adolescent psychiatry department | Steinhövelstraße 5 89075 Ulm |
| Univ.-Prof. Dr. Stephan Heinzel | Free University of Berlin  Professor at the department of Clinical psychology and psychotherapy | Habelschwerdter Allee 45  14195 Berlin |
| Apl. Prof. Dr. Gerhard W. Eschweiler | University Hospital Tuebingen. Senior physician at the department of Psychiatry and Psychotherapy | Calwerstraße 14  72076 Tuebingen |

*Item 21b: Description of any interim analyses and stopping guidelines, including who will have access to these interim results and make the final decision to terminate the trial.*

There will be no interim analysis

## Harms

*Item 22: Plans for collecting, assessing, reporting, and managing solicited and spontaneously reported adverse events and other unintended effects of trial interventions or trial conduct.*

Adjustments to the study or discontinuation of the entire study will be discussed regularly with the independent Data Safety and Monitoring Board in light of reported Adverse Events (AE) or Serious Adverse Events (SAE).

Adverse events (AE) describe any deterioration in the mental or physical condition or behavior of a participant administered the intervention, including events not necessarily caused by or related to the intervention. Following this definition, the following categories are defined as AE for the purposes of this project:

- Onset of new psychological symptoms
- Significant worsening of pre-existing psychological symptoms
- Occurrence of new physical symptoms
- Significant worsening of pre-existing physical symptoms
- Injuries that result in not being able to perform any sports activity for at least 2 months
- Covid-19 infections

A serious adverse event (SAE) is any adverse event that results in death, is life-threatening, requires inpatient hospitalization or prolongation of an existing hospital stay, or results in persistent or significant disability/incapacity. Other significant events may also be considered serious if they endanger the participant or require interventions to prevent any of the above mentioned consequences. Consequently, the following events are considered SAE under this project:

- Suicide
- Suicide attempt
- Other event that resulted in death
- Intentional serious self-injury that resulted in an inpatient stay
- Third party injury
- Event that is acutely life-threatening (i.e., study participant is in acute danger of death)
- Event that results in significant physical disability
- Hospitalization due to psychiatric and somatic symptoms

The above-mentioned categories of AE are recorded during the structured assessments (post, follow-up) via online questionnaires. In addition, participants and therapists (in the case of spontaneous reports by patients) have the opportunity to report AEs to a study telephone (available daily from 9 a.m. to 5 p.m.) at the central office at the University of Tuebingen. Employees of the study center document AEs in a CRF.

Based on the above list, SAEs are recorded by the exercise therapists during the intervention phase and reported to employees of the central office of the University of Tuebingen. In addition, SAEs will be collected by employees of the University of Tuebingen at all three measurement time points (pre, post, follow-up) for the intervention and control groups. They then report SAEs to the study director (Dr. Sebastian Wolf), who consults an independent Data and Safety Monitoring Board for further action within 12 hours after the SAE. The Data Safety and Monitoring Board is composed of independent researchers who are not associated with the project but have expertise in clinical or medical research. All members of the Data Safety and Monitoring Board are listed above. The Data Safety and Monitoring Board decides within 24 hours on any actions that may be necessary (e.g., changes in the trial or similar) and passes these on to the study director. All information on AEs and SAEs will also be recorded in the RedCAP system, like all other data collected.

The following data is documented for each AE/SAE:

- Pseudonym of participant
- Study site to which the participant was associated
- Date of start of SAE and associated study phase (pre, intervention phase, post or follow-up)
- Description of the SAE
- information on whether there was a risk for other participants
- Severity (rated by PI and DSMB)
  - **Mild** – Events require minimal or no treatment and do not interfere with the participant’s daily activities.
  - **Moderate** – Events result in a low level of inconvenience or concern with the therapeutic measures. Moderate events may cause some interference with functioning.
  - **Severe** – Events interrupt a participant’s usual daily activity and may require systemic drug therapy or other treatment. Severe events are usually potentially life-threatening or incapacitating. Of note, the term “severe” does not necessarily equate to “serious”.
- relationship to study intervention (rated by PI and DSMB)
  - **Related** – The AE is known to occur with the study procedures, there is a reasonable possibility that the study procedures caused the AE, or there is a temporal relationship between the study procedures and the event. Reasonable possibility means that there is evidence to suggest a causal relationship between the study procedures and the AE.
  - **Not Related** – There is not a reasonable possibility that the study procedures caused the event, there is no temporal relationship between the study procedures and event onset, or an alternate etiology has been established.
- expectedness
  - as rated by the Data Safety and Monitoring Board
- reporting events to participants
  - yes/no
- Date SAE ended
- Status at end of SAE (resolved, improved, not improved, consequences/type of harm)

## Auditing

*Item 23: Frequency and procedures for auditing trial conduct, if any, and whether the process will be independent from investigators and the sponsor.*

Data monitoring is ensured through a combination of automatic electronic validation through REDCap and manual checks, performed by the evaluating site (LMU). A complete audit trail including any data entry, access, modification, timepoint of randomization, user rights and exports is maintained by REDCap, and only personal information (E-mail) will be deleted from the logs after Data collection. A site initiation procedure for each site was conducted by the LMU before data collection

## Ethics and dissemination

### Research Ethics Approval

*Item 24: Plans for seeking research ethics committee/institutional review board (REC/IRB) approval.*
Ethics approval has been obtained (ID: 888/2020B01, 02/11/2020).

### Protocol Amendments

*Item 25: Plans for communicating important protocol modifications (e.g., changes to eligibility criteria, outcomes, analyses) to relevant parties (e.g., investigators, REC/IRBs, trial participants, trial registries, journals, regulators).*

All protocol modifications will be updated as different versions. Trial registry will be updated continuously. Fundamental changes, such as changing study sites, adaptations of the evaluation concept need to be approved by the funder. All cooperation partners need to agree to fundamental methodological changes. Therefore, an evaluation committee has been installed.

### Consent Or Assent

Item 26a: Who will obtain informed consent or assent from potential trial participants or authorised surrogates, and how (see Item 32).

Interested patients are invited to a first meeting in their respective study center. In this meeting a staff member of the central office from Tuebingen informs patients about the study. All questions are clarified. Afterwards patients sign informed consent. The informed consent files are stored in lockable filing cabinets separated from all other pseudonymized study data. Only selected staff members have access to these cabinets.

Item 26b: Additional consent provisions for collection and use of participant data and biological specimens in ancillary studies, if applicable.

### Confidentiality

*Item 27: How personal information about potential and enrolled participants will be collected, shared, and maintained in order to protect confidentiality before, during, and after the trial.*

### In accordance with the Record of processing activities and data protection concept, participants personal information is collected by recruiting staff at Tuebingen, who input pseudonymized records and researcher-collected data into REDCap and maintain a code list. The only exception is email address, which is also input into REDCap, but which is not accessible to LMU data analysis personnel. At the same time, survey responses are not accessible to recruiting staff. Email addresses will be deleted from the dataset after data collection is completed, and the code list will be deleted after 10 years. Final published datasets will be anonymized, i.e. published without pseudonym.

### Group session videos for process evaluation are stored separately in order to be rated regarding manual adherence. Individual participants are not identified or rated and videos are stored safely at the conducting institution and will be deleted from REDCap after rating.

### Personal information of the diagnostics is stored safely and pseudonymized in files at the Tue-CO.

### ”Declaration Of Interests

*Item 28: Financial and other competing interests for principal investigators for the overall trial and each study site.*

There are no financial or competing interests to declare.

### Access To Data

*Item 29: Statement of who will have access to the final trial dataset, and disclosure of contractual agreements that limit such access for investigators.*

The principal investigator (Sebastian Wolf) and the member of the evaluation team (Keisuke Takano, Eva Herzog, Thomas Ehring) will have access to the complete final data. In addition, all other investigators involved in the project will have access to the final data for pre-specified analyses.

### Ancillary And Post-Trial Care

Item 30: Provisions, if any, for ancillary and post-trial care, and for compensation to those who suffer harm from trial participation.

No provisions and post-trial care are provided.

### Dissemination policy

*Item 31a: Plans for investigators and sponsor to communicate trial results to participants, healthcare professionals, the public, and other relevant groups (e.g., via publication, reporting in results databases, or other data sharing arrangements), including any publication restrictions.*

*Item 31b: Authorship eligibility guidelines and any intended use of professional writers.*

A publication committee that consists of representatives of all partners develops a dissemination policy.

The study PI proposes potential papers. PhD students or other researcher propose a topic/paper and write an abstract to the committee. The committee finally decides about acceptance and authorships.

Item 31c: Plans, if any, for granting public access to the full protocol, participant-level dataset, and statistical code.

# Individual participant data that underlie the results reported in this article will be published after deidentification: text, tables, figures and appendices. Documents that will be further shared: Study protocol, statistical analysis plan, analytic code, aggregated individual study data. Routine/administrative data from health insurances will not be made available. Anyone who wishes to have access the data will have access to the data. Analytic code and aggregated individual study data will be made available on an online repository immediately after publication (or within the peer review process). Participants give informed consent to publish their data after deidentification (despite the routine/administrative data from the health insurances).

# Appendices

Item 32: Model consent form and other related documentation given to participants and authorised surrogates.

Not applicable. Can be sent upon request in German language

# References

(BPtK)], F. C. o. P. B. (2018). *Federal Chamber of Psychotherapists [Bundespsychotherapeutenkammer (BPtK)]. One year after the reform of the psychotherapy guidelines in GErmany [Ein Jahr nach der Reform der Psychotherapie-Richtlinie]*. Retrieved Accessed 08 August 2021 from <https://www.bptk.de/wp-content/uploads/2019/01/20180411_bptk_studie_wartezeiten_2018.pdf>

Abraham, C., Kelly, M. P., West, R., & Michie, S. (2009). The UK National Institute for Health and Clinical Excellence public health guidance on behaviour change: a brief introduction. *Psychol Health Med*, *14*(1), 1-8. <https://doi.org/10.1080/13548500802537903>

Anastasopoulou, P., Tansella, M., Stumpp, J., Shammas, L., & Hey, S. (2012). Classification of human physical activity and energy expenditure estimation by accelerometry and barometry. *Annu Int Conf IEEE Eng Med Biol Soc*, *2012*, 6451-6454. <https://doi.org/10.1109/EMBC.2012.6347471>

Andreu, Y., Galdon, M. J., Dura, E., Ferrando, M., Murgui, S., Garcia, A., & Ibanez, E. (2008). Psychometric properties of the Brief Symptoms Inventory-18 (Bsi-18) in a Spanish sample of outpatients with psychiatric disorders. *Psicothema*, *20*(4), 844-850. <https://www.ncbi.nlm.nih.gov/pubmed/18940093>

Ashdown-Franks, G., Firth, J., Carney, R., Carvalho, A. F., Hallgren, M., Koyanagi, A., Rosenbaum, S., Schuch, F. B., Smith, L., Solmi, M., Vancampfort, D., & Stubbs, B. (2020). Exercise as Medicine for Mental and Substance Use Disorders: A Meta-review of the Benefits for Neuropsychiatric and Cognitive Outcomes. *Sports Med*, *50*(1), 151-170. <https://doi.org/10.1007/s40279-019-01187-6>

Aylett, E., Small, N., & Bower, P. (2018). Exercise in the treatment of clinical anxiety in general practice - a systematic review and meta-analysis. *BMC Health Serv Res*, *18*(1), 559. <https://doi.org/10.1186/s12913-018-3313-5>

Backhaus, J., Junghanns, K., Broocks, A., Riemann, D., & Hohagen, F. (2002). Test-retest reliability and validity of the Pittsburgh Sleep Quality Index in primary insomnia. *J Psychosom Res*, *53*(3), 737-740. <https://doi.org/10.1016/s0022-3999(02)00330-6>

Balkin, R. S., & Lenz, A. S. (2021). Contemporary Issues in Reporting Statistical, Practical, and Clinical Significance in Counseling Research. *Journal of Counseling and Development*, *99*(2), 227-237. <https://doi.org/10.1002/jcad.12370>

Banno, M., Harada, Y., Taniguchi, M., Tobita, R., Tsujimoto, H., Tsujimoto, Y., Kataoka, Y., & Noda, A. (2018). Exercise can improve sleep quality: a systematic review and meta-analysis. *PeerJ*, *6*, e5172. <https://doi.org/10.7717/peerj.5172>

Beesdo-Baum, K., Zaudig, M., & Wittchen, H.-U. (2019). *SCID-5-CV. Strukturiertes Klinisches Interview für DSM-5®-Störungen – Klinische Version Deutsche Bearbeitung des Structured Clinical Interview for DSM-5® Disorders – Clinician Version von Michael B. First, Janet B.W. Williams, Rhonda S. Karg, Robert L. Spitzer*. Hogrefe.

Bilbao, A., Martin-Fernandez, J., Garcia-Perez, L., Mendezona, J. I., Arrasate, M., Candela, R., Acosta, F. J., Estebanez, S., & Retolaza, A. (2021). Psychometric properties of the EQ-5D-5L in patients with major depression: factor analysis and Rasch analysis. *J Ment Health*, 1-11. <https://doi.org/10.1080/09638237.2021.1875422>

Boehm, K. (2021). Gesundheit [Health]. *Data Report 2021 [Datenreport 2021]*, 325-345. Retrieved 2021/05/21, from <https://www.destatis.de/DE/Service/Statistik-Campus/Datenreport/Downloads/datenreport-2021-kap-9.pdf?__blob=publicationFile>

Borg, G. A. (1982). Psychophysical bases of perceived exertion. *Med Sci Sports Exerc*, *14*(5), 377-381. <https://www.ncbi.nlm.nih.gov/pubmed/7154893>

Breyer, B., & Bluemke, M. (2016). *German Version of the Positive and Negative Affect Schedule PANAS (GESIS Panel)*. GESIS - Leibniz-Institut für Sozialwissenschaften. <https://doi.org/10.6102/zis242>

Broocks, A., Bandelow, B., Pekrun, G., George, A., Meyer, T., Bartmann, U., Hillmer-Vogel, U., & Ruther, E. (1998). Comparison of aerobic exercise, clomipramine, and placebo in the treatment of panic disorder. *Am J Psychiatry*, *155*(5), 603-609. <https://doi.org/10.1176/ajp.155.5.603>

Buysse, D. J., Reynolds, C. F., 3rd, Monk, T. H., Berman, S. R., & Kupfer, D. J. (1989). The Pittsburgh Sleep Quality Index: a new instrument for psychiatric practice and research. *Psychiatry Res*, *28*(2), 193-213. <https://doi.org/10.1016/0165-1781(89)90047-4>

Carr, S., Burke, A., Chater, A. M., Howlett, N., & Jones, A. (2021). An Evolving Model of Best Practice in a Community Physical Activity Program: A Case Study of “Active Herts”. *J Phys Act Health*, *18*(12), 1555-1562.

Caspersen, C. J., Powell, K. E., & Christenson, G. M. (1985). Physical activity, exercise, and physical fitness: definitions and distinctions for health-related research. *Public Health Rep*, *100*(2), 126-131.

Coles, M. E., Schubert, J. R., & Nota, J. A. (2015). Sleep, Circadian Rhythms, and Anxious Traits. *Curr Psychiatry Rep*, *17*(9), 73. <https://doi.org/10.1007/s11920-015-0613-x>

Crone, D., Johnston, L. H., Gidlow, C., Henley, C., & James, D. V. B. (2008). Uptake and Participation in Physical Activity Referral Schemes in the UK: An Investigation of Patients Referred with Mental Health Problems. *Issues Ment Health Nurs*, *29*(10), 1088-1097. <https://doi.org/10.1080/01612840802319837>

De Hert, M., Detraux, J., & Vancampfort, D. (2018). The intriguing relationship between coronary heart disease and mental disorders. *Dialogues Clin Neurosci*, *20*(1), 31-40.

Derogatis, L. R., & Fitzpatrick, M. (2004). The SCL-90-R, the Brief Symptom Inventory (BSI), and the BSI-18. In *The use of psychological testing for treatment planning and outcomes assessment: Instruments for adults, Volume 3, 3rd ed.* (pp. 1-41). Lawrence Erlbaum Associates Publishers.

Destatis [Statistisches Bundesamt]. (2015, 2021/08/09). *Destatis [Statistisches Bundesamt]. Health Costs [Gesundheistkosten]*. Retrieved Accessed 09 August 2021 from <https://www.destatis.de/DE/Themen/Gesellschaft-Umwelt/Gesundheit/Krankheitskosten/Tabellen/krankheitsklassen-alter.html;jsessionid=7FF730CB0621F34573C01BA9DF4766BC.live731>.

Deutsche Rentenversicherung Bund (DRV). (2014). *Strukturqualität von Reha-Einrichungen - Anforderungen der Deutschen Rentenversicherung (Structural quality of rehabilitation and medical care facilities - requirements of the German Pension Insurance)* (2. überarbeitete und erweiterte Auflage ed.). DRV.

Dieck, A., Morin, C. M., & Backhaus, J. (2018). A German version of the Insomnia Severity Index. *Somnologie*, *22*(1), 27-35. <https://doi.org/10.1007/s11818-017-0147-z>

Ehring, T., Zetsche, U., Weidacker, K., Wahl, K., Schonfeld, S., & Ehlers, A. (2011). The Perseverative Thinking Questionnaire (PTQ): Validation of a Content-Independent Measure of Repetitive Negative Thinking. *J Behav Ther Exp Psychiatry*, *42*(2), 225-232. <https://doi.org/10.1016/j.jbtep.2010.12.003>

Ellard, D. R., Thorogood, M., Underwood, M., Seale, C., & Taylor, S. J. C. (2014). Whole home exercise intervention for depression in older care home residents (the OPERA study): a process evaluation. *BMC Medicine*, *12*(1), 1. <https://doi.org/10.1186/1741-7015-12-1>

Esquivel, G., Diaz-Galvis, J., Schruers, K., Berlanga, C., Lara-Munoz, C., & Griez, E. (2008). Acute exercise reduces the effects of a 35% CO2 challenge in patients with panic disorder. *J Affect Disord*, *107*(1-3), 217-220. <https://doi.org/10.1016/j.jad.2007.07.022>

Farrand, P., Pentecost, C., Greaves, C., Taylor, R. S., Warren, F., Green, C., Hillsdon, M., Evans, P., Welsman, J., & Taylor, A. H. (2014). A written self-help intervention for depressed adults comparing behavioural activation combined with physical activity promotion with a self-help intervention based upon behavioural activation alone: study protocol for a parallel group pilot randomised controlled trial (BAcPAc). *Trials*, *15*(1), 196. <https://doi.org/10.1186/1745-6215-15-196>

Faul, F., Erdfelder, E., Buchner, A., & Lang, A.-G. (2009). Statistical Power Analyses Using G*Power 3.1: Tests for Correlation and Regression Analyses. *Behav Res Methods*, *41*, 1149-1160. <https://doi.org/10.3758/BRM.41.4.1149>

Faul, F., Erdfelder, E., Lang, A.-G., & Buchner, A. J. B. R. M. (2007). G*Power 3: A flexible statistical power analysis program for the social, behavioral, and biomedical sciences [journal article]. *39*(2), 175-191. <https://doi.org/10.3758/bf03193146>

Federal Chamber of Psychotherapists [Bundespsychotherapeutenkammer (BPtK)]. (2021, 2021/03/29). *Federal Chamber of Psychotherapists [Bundespsychotherapeutenkammer (BPtK)]. Months of waiting time for psychotherapy [BPtK-Auswertung: Monatelange Wartezeiten bei Psychotherapeut*innen]*. Retrieved Accessed 08 August 2021 from <https://www.bptk.de/wp-content/uploads/2021/03/20210329_pm_bptk_monatelange-Wartezeiten.pdf>.

Federal Institute for Occupational Safety and Health [Bundesanstalt für Arbeitsschutz und Arbeitsmedizin (BAuA)]. (2021). *Federal Institute for Occupational Safety and Health [Bundesanstalt für Arbeitsschutz und Arbeitsmedizin (BAuA)]. Economic costs due to Incapacity to work 2019 [Volkswirtschaftliche Kosten durch Arbeitsunfähigkeit 2019]*. Retrieved Accessed 08 August 2021 from <https://www.baua.de/DE/Themen/Arbeitswelt-und-Arbeitsschutz-im-Wandel/Arbeitsweltberichterstattung/Kosten-der-AU/pdf/Kosten-2019.pdf?__blob=publicationFile&v=3>.

Finger, J. D., Tafforeau, J., Gisle, L., Oja, L., Ziese, T., Thelen, J., Mensink, G. B., & Lange, C. (2015). Development of the European Health Interview Survey - Physical Activity Questionnaire (EHIS-PAQ) to monitor physical activity in the European Union. *Arch Public Health*, *73*, 59. <https://doi.org/10.1186/s13690-015-0110-z>

Fiuza-Luces, C., Garatachea, N., Berger, N., & Lucia, A. (2013). Exercise is the real polypill. *Physiology*, *28*(5), 330-358. <https://doi.org/10.1152/physiol.00019.2013>

Ford, I., & Norrie, J. (2016). Pragmatic Trials. *N Engl J Med*, *375*(5), 454-463. <https://doi.org/10.1056/NEJMra1510059>

Fuchs, R., Klaperski, S., Gerber, M., & Seelig, H. (2015). Messung der Bewegungs- und Sportaktivität mit dem BSA-Fragebogen. *Zeitschrift für Gesundheitspsychologie*, *23*(2), 60-76. <https://doi.org/10.1026/0943-8149/a000137>

Fuchs, R., Seelig, H., Gohner, W., Burton, N. W., & Brown, W. J. (2012). Cognitive mediation of intervention effects on physical exercise: causal models for the adoption and maintenance stage. *Psychol Health*, *27*(12), 1480-1499. <https://doi.org/10.1080/08870446.2012.695020>

Fynn, J. F., Hardeman, W., Milton, K., & Jones, A. P. (2020). A Scoping Review of Evaluation Frameworks and their Applicability to Real-World Physical Activity and Dietary Change Programme Evaluation. *BMC Public Health*, *20*(1), 1000-1016. <https://doi.org/10.1186/s12889-020-09062-0>

Garber, C. E., Blissmer, B., Deschenes, M. R., Franklin, B. A., Lamonte, M. J., Lee, I. M., Nieman, D. C., Swain, D. P., & American College of Sports, M. (2011). American College of Sports Medicine position stand. Quantity and quality of exercise for developing and maintaining cardiorespiratory, musculoskeletal, and neuromotor fitness in apparently healthy adults: guidance for prescribing exercise. *Med Sci Sports Exerc*, *43*(7), 1334-1359. <https://doi.org/10.1249/MSS.0b013e318213fefb>

Gaudlitz, K., Plag, J., Dimeo, F., & Strohle, A. (2015). Aerobic exercise training facilitates the effectiveness of cognitive behavioral therapy in panic disorder. *Depress Anxiety*, *32*(3), 221-228. <https://doi.org/10.1002/da.22337>

Geidl, W., Deprins, J., Streber, R., Rohrbach, N., Sudeck, G., & Pfeifer, K. (2018). Exercise Therapy in Medical Rehabilitation: Study Protocol of a National Survey at Facility and Practitioner Level with a Mixed Method Design. *Contemp Clin Trials Commun*, *11*, 37-45. <https://doi.org/10.1016/j.conctc.2018.05.004>

Geidl, W., Hofmann, J., Gohner, W., Sudeck, G., & Pfeifer, K. (2012). Behaviour-orientated exercise therapy--initiating and maintaining a physically active lifestyle. *Rehabilitation (Stuttg)*, *51*(4), 259-268. <https://doi.org/10.1055/s-0031-1280803> (Verhaltensbezogene Bewegungstherapie--Bindung an einen korperlich aktiven Lebensstil.)

GKV Spitzenverband. *Gemeinsame Rahmenempfehlung für ambulante und stationäre Vorsorge- und Rehabilitationsleistungen auf der Grundlage des § 111a SGB V bzw. §§ 40 + 43 SGB V (Joint framework recommendation for outpatient and inpatient preventive and rehabilitation services)*. GKV Spitzenverband.

Global Burden of Disease Collaborative Network. (2020, 24 May 2021). *Global Burden of Disease Collaborative Network. Global Burden of Disease Study 2019 (GBD 2019) Results*. Institute for Health Metrics and Evaluation (IHME). <http://ghdx.healthdata.org/gbd-results-tool>. Accessed 09 August 2021

Göhner, W., Schagg, D., Küffner, R., & Reusch, A. (2018). Psychologische Strategien zur Bewegungsförderung: Entwicklung von Fortbildungen für die Bewegungstherapie (BeFo) (Psychological Strategies for Promoting Physical Activity: Development of Further Training Measures for Physical Exercise Therapy). *B&G Bewegungstherapie und Gesundheitssport (B&G Physical exercise therapy and health sports)*, *34*(4), 168-177. <https://doi.org/10.1055/a-0641-8776>

Goldsmith, K. A., MacKinnon, D. P., Chalder, T., White, P. D., Sharpe, M., & Pickles, A. (2018). Tutorial: The practical application of longitudinal structural equation mediation models in clinical trials. *Psychol Methods*, *23*(2), 191-207. <https://doi.org/10.1037/met0000154>

Goldstein, L. A., Mehling, W. E., Metzler, T. J., Cohen, B. E., Barnes, D. E., Choucroun, G. J., Silver, A., Talbot, L. S., Maguen, S., Hlavin, J. A., Chesney, M. A., & Neylan, T. C. (2018). Veterans Group Exercise: A randomized pilot trial of an Integrative Exercise program for veterans with posttraumatic stress. *J Affect Disord*, *227*, 345-352. <https://doi.org/10.1016/j.jad.2017.11.002>

Gratz, K. L., & Roemer, L. (2004). Multidimensional Assessment of Emotion Regulation and Dysregulation: Development, Factor Structure, and Initial Validation of the Difficulties in Emotion Regulation Scale. *J Psychopathol Behav*, *26*(1), 41-54. <https://doi.org/10.1023/B:JOBA.0000007455.08539.94>

Hajak, G. (2001). Epidemiology of severe insomnia and its consequences in Germany. *Eur Arch Psychiatry Clin Neurosci*, *251*(2), 49-56. <https://doi.org/10.1007/s004060170052>

Hall, M., Thayer, J. F., Germain, A., Moul, D., Vasko, R., Puhl, M., Miewald, J., & Buysse, D. J. (2007). Psychological stress is associated with heightened physiological arousal during NREM sleep in primary insomnia. *Behav Sleep Med*, *5*(3), 178-193. <https://doi.org/10.1080/15402000701263221>

Harmsen, M., Peters, M., & Wensing, M. (2005). Barriers and Facilitators Assessment Instrument Introduction, Instructions and Instrument. *IQ healthcare Scientific Institute for Quality of Healthcare. Radboud University Medical Center Nijmegen*.

Harris, P. A., Taylor, R., Minor, B. L., Elliott, V., Fernandez, M., O'Neal, L., McLeod, L., Delacqua, G., Delacqua, F., Kirby, J., Duda, S. N., & Consortium, R. E. (2019). The REDCap consortium: Building an international community of software platform partners. *J Biomed Inform*, *95*, 103208. <https://doi.org/10.1016/j.jbi.2019.103208>

Harris, P. A., Taylor, R., Thielke, R., Payne, J., Gonzalez, N., & Conde, J. G. (2009). Research electronic data capture (REDCap)--a metadata-driven methodology and workflow process for providing translational research informatics support. *J Biomed Inform*, *42*(2), 377-381. <https://doi.org/10.1016/j.jbi.2008.08.010>

Harvey, A. G. (2002). A cognitive model of insomnia. *Behav Res Ther*, *40*(8), 869-893. <https://doi.org/10.1016/s0005-7967(01)00061-4>

Herdman, M., Gudex, C., Lloyd, A., Janssen, M., Kind, P., Parkin, D., Bonsel, G., & Badia, X. (2011). Development and preliminary testing of the new five-level version of EQ-5D (EQ-5D-5L). *Qual Life Res*, *20*(10), 1727-1736. <https://doi.org/10.1007/s11136-011-9903-x>

Hinz, A., Klein, A. M., Brahler, E., Glaesmer, H., Luck, T., Riedel-Heller, S. G., Wirkner, K., & Hilbert, A. (2017). Psychometric evaluation of the Generalized Anxiety Disorder Screener GAD-7, based on a large German general population sample. *J Affect Disord*, *210*, 338-344. <https://doi.org/10.1016/j.jad.2016.12.012>

Hinz, A., Kohlmann, T., Stobel-Richter, Y., Zenger, M., & Brahler, E. (2014). The quality of life questionnaire EQ-5D-5L: psychometric properties and normative values for the general German population. *Qual Life Res*, *23*(2), 443-447. <https://doi.org/10.1007/s11136-013-0498-2>

Hoffmann, T., Glasziou, P., Boutron, I., Milne, R., Perera, R., Moher, D., Altman, D., Barbour, V., Macdonald, H., Johnston, M., Lamb, S. E., Dixon-Woods, M., McCulloch, P., Wyatt, J., Chan Phelan, A. W., & Michie, S. (2016). *Die TIDieR Checkliste und Anleitung – ein Instrument für eine verbesserte Interventionsbeschreibung und Replikation* (Vol. 78). <https://doi.org/10.1055/s-0041-111066>

Iancu, I., Bodner, E., & Ben-Zion, I. Z. (2015). Self esteem, dependency, self-efficacy and self-criticism in social anxiety disorder. *Compr Psychiatry*, *58*, 165-171. <https://doi.org/10.1016/j.comppsych.2014.11.018>

Jacobson, N. S., Follette, W. C., & Revenstorf, D. (1984). Psychotherapy outcome research: Methods for reporting variability and evaluating clinical significance. *Behavior Therapy*, *15*(4), 336-352. <https://doi.org/10.1016/s0005-7894(84)80002-7>

Jacobson, N. S., & Truax, P. (1992). Clinical significance : A statistical approach to defining meaningful change in psychotherapy research. In *Methodological issues & strategies in clinical research.* (pp. 631-648). American Psychological Association. <https://doi.org/10.1037/10109-042>

Jacquart, J., Dutcher, C. D., Freeman, S. Z., Stein, A. T., Dinh, M., Carl, E., & Smits, J. A. J. (2019). The effects of exercise on transdiagnostic treatment targets: A meta-analytic review. *Behav Res Ther*, *115*, 19-37. <https://doi.org/10.1016/j.brat.2018.11.007>

Jarbin, H., Höglund, K., Skarphedinsson, G., & Bremander, A. (2021). Aerobic Exercise for Adolescent Outpatients with Persistent Major Depression: Feasibility and Acceptability of Moderate to Vigorous Group Exercise in a Clinically Referred Sample. *Clin Child Psychol Psychiatry*, *26*(4), 954-967. <https://doi.org/10.1177/13591045211000782>

Johnson, L. A., & Caldwell, B. E. (2011). Race, Gender, and Therapist Confidence: Effects on Satisfaction with the Therapeutic Relationship in MFT. *Am J of Fam Ther*, *39*(4), 307-324. <https://doi.org/10.1080/01926187.2010.532012>

Kendler, K. S., Karkowski, L. M., & Prescott, C. A. (1999). Causal relationship between stressful life events and the onset of major depression. *Am J Psychiatry*, *156*(6), 837-841. <https://doi.org/10.1176/ajp.156.6.837>

Kessler, R. C., Chiu, W. T., Demler, O., Merikangas, K. R., & Walters, E. E. (2005). Prevalence, severity, and comorbidity of 12-month DSM-IV disorders in the National Comorbidity Survey Replication. *Arch Gen Psychiatry*, *62*(6), 617-627. <https://doi.org/10.1001/archpsyc.62.6.617>

Kien, C., Griebler, U., Schultes, M.-T., Thaler, K. J., & Stamm, T. (2021). Psychometric testing of the German versions of three implementation outcome measures. *Glob Implement Res App*, *1*(3), 183-194.

Kocalevent, R. D., Hinz, A., & Brahler, E. (2013). Standardization of the depression screener patient health questionnaire (PHQ-9) in the general population. *Gen Hosp Psychiatry*, *35*(5), 551-555. <https://doi.org/10.1016/j.genhosppsych.2013.04.006>

Kramer, L. V., Helmes, A. W., Seelig, H., Fuchs, R., & Bengel, J. (2014). Correlates of reduced exercise behaviour in depression: the role of motivational and volitional deficits. *Psychol Health*, *29*(10), 1206-1225. <https://doi.org/10.1080/08870446.2014.918978>

Kroenke, K., Spitzer, R. L., & Williams, J. B. (2001). The PHQ-9: validity of a brief depression severity measure. *J Gen Intern Med*, *16*(9), 606-613. <https://doi.org/10.1046/j.1525-1497.2001.016009606.x>

Kroenke, K., Spitzer, R. L., Williams, J. B., & Lowe, B. (2010). The Patient Health Questionnaire Somatic, Anxiety, and Depressive Symptom Scales: a systematic review. *Gen Hosp Psychiatry*, *32*(4), 345-359. <https://doi.org/10.1016/j.genhosppsych.2010.03.006>

Kroenke, K., Spitzer, R. L., Williams, J. B., Monahan, P. O., & Lowe, B. (2007). Anxiety disorders in primary care: prevalence, impairment, comorbidity, and detection. *Ann Intern Med*, *146*(5), 317-325. <https://doi.org/10.7326/0003-4819-146-5-200703060-00004>

Kruger-Gottschalk, A., Knaevelsrud, C., Rau, H., Dyer, A., Schafer, I., Schellong, J., & Ehring, T. (2017). The German version of the Posttraumatic Stress Disorder Checklist for DSM-5 (PCL-5): psychometric properties and diagnostic utility. *BMC Psychiatry*, *17*(1), 379. <https://doi.org/10.1186/s12888-017-1541-6>

Kuckartz, U. (2016). *Qualitative Inhaltsanalyse. Methoden, Praxis, Computerunterstützung (Qualitative content analysis. Methods, practice, computer support)* (Vol. 3). Beltz.

Lee, H., Herbert, R. D., & McAuley, J. H. (2019). Mediation Analysis. *JAMA*, *321*(7), 697-698. <https://doi.org/10.1001/jama.2018.21973>

Legrand, F. D., & Neff, E. M. (2016). Efficacy of exercise as an adjunct treatment for clinically depressed inpatients during the initial stages of antidepressant pharmacotherapy: An open randomized controlled trial. *J Affect Disord*, *191*, 139-144. <https://doi.org/10.1016/j.jad.2015.11.047>

Levis, B., Benedetti, A., & Thombs, B. D. (2019). Accuracy of Patient Health Questionnaire-9 (PHQ-9) for screening to detect major depression: individual participant data meta-analysis. *BMJ*, *365*, l1476. <https://doi.org/10.1136/bmj.l1476>

Liang, J., Xian, D., Liu, X., Fu, J., Zhang, X., Tang, B., & Lei, J. (2018). Usability Study of Mainstream Wearable Fitness Devices: Feature Analysis and System Usability Scale Evaluation. *JMIR mHealth uHealth*, *6*(11), e11066. <https://doi.org/10.2196/11066>

Lowe, B., Decker, O., Muller, S., Brahler, E., Schellberg, D., Herzog, W., & Herzberg, P. Y. (2008). Validation and standardization of the Generalized Anxiety Disorder Screener (GAD-7) in the general population. *Med Care*, *46*(3), 266-274. <https://doi.org/10.1097/MLR.0b013e318160d093>

Maciejewski, P. K., Prigerson, H. G., & Mazure, C. M. (2000). Self-efficacy as a mediator between stressful life events and depressive symptoms. Differences based on history of prior depression. *Br J Psychiatry*, *176*, 373-378. <https://doi.org/10.1192/bjp.176.4.373>

Messner, E.-M., Terhorst, Y., Barke, A., Baumeister, H., Stoyanov, S., Hides, L., Kavanagh, D., Pryss, R., Sander, L., & Probst, T. (2020). The German Version of the Mobile App Rating Scale (MARS-G): Development and Validation Study. *JMIR mHealth uHealth*, *8*(3), e14479. <https://doi.org/10.2196/14479>

Michie, S., Ashford, S., Sniehotta, F. F., Dombrowski, S. U., Bishop, A., & French, D. P. (2011). A refined taxonomy of behaviour change techniques to help people change their physical activity and healthy eating behaviours: the CALO-RE taxonomy. *Psychol Health*, *26*(11), 1479-1498. <https://doi.org/10.1080/08870446.2010.540664>

Modgill, G., Patten, S. B., Knaak, S., Kassam, A., & Szeto, A. C. H. (2014). Opening Minds Stigma Scale for Health Care Providers (OMS-HC): Examination of Psychometric Properties and Responsiveness. *BMC psychiatry*, *14*(1), 120-130. <https://doi.org/10.1186/1471-244X-14-120>

Moore, G., Audrey, S., Barker, M., Bond, L., Bonell, C., Hardeman, W., Moore, L., O’Cathain, A., Tinati, T., Wight, D., & Baird, J. (2015). Process Evaluation of Complex Interventions: Medical Research Council Guidance. *BMJ*, *350*, h1258. <https://doi.org/10.1136/bmj.h1258>

Moreno-Peral, P., Conejo-Ceron, S., Motrico, E., Rodriguez-Morejon, A., Fernandez, A., Garcia-Campayo, J., Roca, M., Serrano-Blanco, A., Rubio-Valera, M., & Bellon, J. A. (2014). Risk factors for the onset of panic and generalised anxiety disorders in the general adult population: a systematic review of cohort studies. *J Affect Disord*, *168*, 337-348. <https://doi.org/10.1016/j.jad.2014.06.021>

Morin, C. M. (1993). *Insomnia: Psychological assessment and management*. Guilford Press.

Morres, I. D., Hatzigeorgiadis, A., Stathi, A., Comoutos, N., Arpin-Cribbie, C., Krommidas, C., & Theodorakis, Y. (2018). Aerobic exercise for adult patients with major depressive disorder in mental health services: A systematic review and meta-analysis. *Depress Anxiety*. <https://doi.org/10.1002/da.22842>

Morres, I. D., Hatzigeorgiadis, A., Stathi, A., Comoutos, N., Arpin-Cribbie, C., Krommidas, C., & Theodorakis, Y. (2019). Aerobic exercise for adult patients with major depressive disorder in mental health services: A systematic review and meta-analysis. *Depress Anxiety*, *36*(1), 39-53. <https://doi.org/10.1002/da.22842>

Morres, I. D., Hinton-Bayre, A., Motakis, E., Carter, T., & Callaghan, P. (2019). A pragmatic randomised controlled trial of preferred intensity exercise in depressed adult women in the United Kingdom: secondary analysis of individual variability of depression. *BMC Public Health*, *19*(1), 941. <https://doi.org/10.1186/s12889-019-7238-7>

Naci, H., & Ioannidis, J. P. (2015). Comparative effectiveness of exercise and drug interventions on mortality outcomes: metaepidemiological study. *Br J Sports Med*, *49*(21), 1414-1422. <https://doi.org/10.1136/bjsports-2015-f5577rep>

Naragon-Gainey, K. (2010). Meta-analysis of the relations of anxiety sensitivity to the depressive and anxiety disorders. *Psychol Bull*, *136*(1), 128-150. <https://doi.org/10.1037/a0018055>

Nübling, R., T, B., Jeschke, K., M, O., Sarubin, N., & Juergen, S. (2014). Care of Mentally Ill Adults in Germany - Need and Utilization as well as Effectiveness and Efficiency of Psychotherapy [Versorgung psychisch kranker Erwachsener in Deutschland - Bedarf und Inanspruchnahme sowie Effektivität und Effizienz von Psychotherapie]. *Psychotherapeutenjournal*, *13*, 389-397.

Oei, T. P., & Green, A. L. (2008). The Satisfaction With Therapy and Therapist Scale--Revised (STTS-R) for Group Psychotherapy: Psychometric Properties and Confirmatory Factor Analysis. *Prof Psychol - Res Pr*, *39*(4), 435-442. <https://doi.org/10.1037/0735-7028.39.4.435>

Olatunji, B. O., & Wolitzky-Taylor, K. B. (2009). Anxiety sensitivity and the anxiety disorders: a meta-analytic review and synthesis. *Psychol Bull*, *135*(6), 974-999. <https://doi.org/10.1037/a0017428>

Orlinsky, D., Ambühl, H., Rønnestad, M. H., Davis, J., Gerin, P., Davis, M., Willutzki, U., Botermans, J.-F., Dazord, A., Cierpka, M., Aapro, N., Buchheim, P., Bae, S., Davidson, C., Friis-Jorgensen, E., Joo, E., Kalmykova, E., Meyerberg, J., Northcut, T., . . . Network, S. C. R. (1999). Development of Psychotherapists: Concepts, Questions, and Methods of a Collaborative International Study. *Psychother Res*, *9*(2), 127-153. <https://doi.org/10.1080/10503309912331332651>

Patel, V. (2012). Global mental health: from science to action. *Harv Rev Psychiatry*, *20*(1), 6-12. <https://doi.org/10.3109/10673229.2012.649108>

Peters, D. H., Adam, T., Alonge, O., Agyepong, I. A., & Tran, N. (2013). Implementation Research: What It Is and How to Do It. *BMJ Brit Med J*, *347*(1), f6753. <https://doi.org/10.1136/bmj.f6753>

Peters, M. A. J., Harmsen, M., Laurant, M. G. H., & Wensing, M. (2002). *Barriers and facilitators assessment instrument*.

Petrides, K. V., & Furnham, A. (2006). The Role of Trait Emotional Intelligence in a Gender-Specific Model of Organizational Variables. *J Appl Soc Psychol*, *36*(2), 552-569. <https://doi.org/10.1111/j.0021-9029.2006.00019.x>

Plummer, F., Manea, L., Trepel, D., & McMillan, D. (2016). Screening for anxiety disorders with the GAD-7 and GAD-2: a systematic review and diagnostic metaanalysis. *Gen Hosp Psychiatry*, *39*, 24-31. <https://doi.org/10.1016/j.genhosppsych.2015.11.005>

Powers, M. B., Medina, J. L., Burns, S., Kauffman, B. Y., Monfils, M., Asmundson, G. J., Diamond, A., McIntyre, C., & Smits, J. A. (2015). Exercise Augmentation of Exposure Therapy for PTSD: Rationale and Pilot Efficacy Data. *Cogn Behav Ther*, *44*(4), 314-327. <https://doi.org/10.1080/16506073.2015.1012740>

R Core, T. (2021). *R: A language and environment for statistical computing. R Foundation for Statistical Computing, Vienna, Austria*. In

Rabe-Menssen, C., Dazer, A., & Maaß, E. (2021). *Report Psychotherapy [Report Psychotherapie 2021]* (2nd ed.). German Psychotherapists Association [Deutsche Psychotherapeuten Vereinigung (DPtV)].

Rehm, J., & Shield, K. D. (2019). Global Burden of Disease and the Impact of Mental and Addictive Disorders. *Curr Psychiatry Rep*, *21*(2), 10. <https://doi.org/10.1007/s11920-019-0997-0>

Reinders, H. (2015). III-5 Interview. In *Empirische Bildungsforschung (educational research)* (pp. 93-107). Springer.

Resnick, B., & Jenkins, L. S. (2000). Testing the Reliability and Validity of the Self-Efficacy for Exercise Scale. *Nursing Res 49*(3), 154-159.

Rigotti, T., Schyns, B., & Mohr, G. (2008). A Short Version of the Occupational Self-Efficacy Scale: Structural and Construct Validity Across Five Countries. *J Career Assessment*, *16*(2), 238-255. <https://doi.org/10.1177/1069072707305763>

Rodgers, W. M., Wilson, P. M., Hall, C. R., Fraser, S. N., & Murray, T. (2008). Evidence for a Multidimensional Self-Efficacy for Exercise Scale. *Res Q Exercise Sport*, *79*(2), 222-234. <https://doi.org/10.1080/02701367.2008.10599485>

Rosenbaum, S., Vancampfort, D., Steel, Z., Newby, J., Ward, P. B., & Stubbs, B. (2015). Physical activity in the treatment of Post-traumatic stress disorder: A systematic review and meta-analysis. *Psychiatry Res*, *230*(2), 130-136. <https://doi.org/10.1016/j.psychres.2015.10.017>

Schneider, E. E., Schönfelder, S., Domke-Wolf, M., & Wessa, M. (2020). Measuring Stress in Clinical and Nonclinical Subjects Using a German Adaptation of the Perceived Stress Scale. *Int J Clin Health Psychol*, *20*(2), 173-181. <https://doi.org/10.1016/j.ijchp.2020.03.004>

Schuch, F., Vancampfort, D., Firth, J., Rosenbaum, S., Ward, P., Reichert, T., Bagatini, N. C., Bgeginski, R., & Stubbs, B. (2017). Physical activity and sedentary behavior in people with major depressive disorder: A systematic review and meta-analysis. *J Affect Disord*, *210*, 139-150. <https://doi.org/10.1016/j.jad.2016.10.050>

Schulte, D. (2005). Messung der Therapieerwartung und Therapieevaluation von Patienten (PATHEV) (Measurement of Patients' Expectation of Therapy and Evaluation of Therapy (PATHEV)). *Z Kl Psych und Psychoth*, *34*(3), 176-187. <https://doi.org/10.1026/1616-3443.34.3.176>

Schulz, H., Nübling, R., & Rüddel, H. (1995). Entwicklung einer Kurzform eines Fragebogens zur Psychotherapiemotivation (Development of a Short Form of a Questionnaire on psychotherapy Motivation). *Verhaltenstherapie*, *5*(2), 89-95. <https://doi.org/10.1159/000258901>

Skivington, K., Matthews, L., Simpson, S. A., Craig, P., Baird, J., Blazeby, J. M., Boyd, K. A., Craig, N., French, D. P., McIntosh, E., Petticrew, M., Rycroft-Malone, J., White, M., & Moore, L. (2021). A New Framework for Developing and Evaluating Complex Interventions: Update of Medical Research Council Guidance. *BMJ*, *374*, n2061. <https://doi.org/10.1136/bmj.n2061>

Slade, S. C., Dionne, C. E., Underwood, M., & Buchbinder, R. (2016). Consensus on Exercise Reporting Template (CERT): Explanation and Elaboration Statement. *Br J Sports Med*, *50*(23), 1428-1437. <https://doi.org/10.1136/bjsports-2016-096651>

Sniehotta, F. F., Schwarzer, R., Scholz, U., & Schüz, B. (2005). Action Planning and Coping Planning for Long-Term Lifestyle Change: Theory and Assessment. *Eur J Soc Psychol*, *35*(4), 565-576. <https://doi.org/10.1002/ejsp.258>

Spitzer, C., Hammer, S., Lowe, B., Grabe, H. J., Barnow, S., Rose, M., Wingenfeld, K., Freyberger, H. J., & Franke, G. H. (2011). [The short version of the Brief Symptom Inventory (BSI -18): preliminary psychometric properties of the German translation]. *Fortschr Neurol Psychiatr*, *79*(9), 517-523. <https://doi.org/10.1055/s-0031-1281602> (Die Kurzform des Brief Symptom Inventory (BSI -18): erste Befunde zu den psychometrischen Kennwerten der deutschen Version.)

Spitzer, R. L., Kroenke, K., & Williams, J. B. (1999). Validation and utility of a self-report version of PRIME-MD: the PHQ primary care study. Primary Care Evaluation of Mental Disorders. Patient Health Questionnaire. *JAMA*, *282*(18), 1737-1744. <https://doi.org/10.1001/jama.282.18.1737>

Spitzer, R. L., Kroenke, K., Williams, J. B., & Lowe, B. (2006). A brief measure for assessing generalized anxiety disorder: the GAD-7. *Arch Intern Med*, *166*(10), 1092-1097. <https://doi.org/10.1001/archinte.166.10.1092>

Steinmayr, R., & Spinath, B. (2010). Konstruktion und erste Validierung einer Skala zur Erfassung subjektiver schulischer Werte (SESSW) (Construction and Initial Validation of a Scale for the Assessment of Subjective School Values (SESSW)). *Diagnostica*, *56*(4), 195-211. <https://doi.org/10.1026/0012-1924/a000023>

Stubbs, B., Vancampfort, D., Hallgren, M., Firth, J., Veronese, N., Solmi, M., Brand, S., Cordes, J., Malchow, B., Gerber, M., Schmitt, A., Correll, C. U., De Hert, M., Gaughran, F., Schneider, F., Kinnafick, F., Falkai, P., Moller, H. J., & Kahl, K. G. (2018). EPA guidance on physical activity as a treatment for severe mental illness: a meta-review of the evidence and Position Statement from the European Psychiatric Association (EPA), supported by the International Organization of Physical Therapists in Mental Health (IOPTMH). *Eur Psychiatry*, *54*, 124-144. <https://doi.org/10.1016/j.eurpsy.2018.07.004>

Sudeck, G., & Pfeifer, K. (2016). Physical Activity-Related Health Competence as an Integrative Objective in Exercise Therapy and Health Sports - Conception and Validation of a Short Questionnaire. *Sportwissenschaft*, *46*(2), 74-87. <https://doi.org/10.1007/s12662-016-0405-4>

Szota, K., Thielemann, J. F. B., Christiansen, H., Rye, M., Aarons, G. A., & Barke, A. (2021). Cross-cultural adaption and psychometric investigation of the German version of the Evidence Based Practice Attitude Scale (EBPAS-36D). *Health Res Policy Syst*, *19*(1), 90. <https://doi.org/10.1186/s12961-021-00736-8>

Thomas, S., Reading, J., & Shephard, R. J. (1992). Revision of the Physical Activity Readiness Questionnaire (PAR-Q). *Can J Sport Sci*, *17*(4), 338-345. <https://www.ncbi.nlm.nih.gov/pubmed/1330274>

Usami, S., Murayama, K., & Hamaker, E. L. (2019). A unified framework of longitudinal models to examine reciprocal relations. *Psychol Methods*, *24*(5), 637-657. <https://doi.org/10.1037/met0000210>

van Dongen, E. J. I., Haveman-Nies, A., Wezenbeek, N. L. W., Dorhout, B. G., Doets, E. L., & de Groot, L. C. P. G. M. (2018). Effect, Process, and Economic Evaluation of a Combined Resistance Exercise and Diet Intervention (ProMuscle in Practice) for Community-Dwelling Older Adults: Design and Methods of a Randomised Controlled Trial. *BMC Public Health*, *18*(1), 877-889. <https://doi.org/10.1186/s12889-018-5788-8>

VERBI, S. (2021). *MAXQDA 2022*. In VERBI Software.

von Brachel, R., Bieda, A., Margraf, J., & Hirschfeld, G. (2020). Longitudinal Measurement Invariance of the Brief Symptom Inventory (BSI)-18 in Psychotherapy Patients. *European Journal of Psychological Assessment*, *36*(1), 12-18. <https://doi.org/10.1027/1015-5759/a000480>

Voorn, E. L., Koopman, F. S., Brehm, M. A., Beelen, A., de Haan, A., Gerrits, K. H. L., & Nollet, F. (2016). Aerobic Exercise Training in Post-Polio Syndrome: Process Evaluation of a Randomized Controlled Trial. *PLoS One*, *11*(7), e0159280. <https://doi.org/10.1371/journal.pone.0159280>

Wahl, K., Ehring, T., Kley, H., Lieb, R., Meyer, A., Kordon, A., Heinzel, C. V., Mazanec, M., & Schonfeld, S. (2019). Is repetitive negative thinking a transdiagnostic process? A comparison of key processes of RNT in depression, generalized anxiety disorder, obsessive-compulsive disorder, and community controls. *J Behav Ther Exp Psychiatry*, *64*, 45-53. <https://doi.org/10.1016/j.jbtep.2019.02.006>

Walburg, F. S., de Joode, J. W., Brandt, H. E., van Tulder, M. W., Adriaanse, M. C., & van Meijel, B. (2022). Implementation of a Lifestyle Intervention for People with a Severe Mental Illness (SMILE): A Process Evaluation. *BMC Health Serv Res*, *22*(1), 27-39. <https://doi.org/10.1186/s12913-021-07391-3>

Wilmers, F., Munder, T., Leonhart, R., Herzog, T., Plassmann, R., Barth, J., & Linster, H. W. (2008). Die deutschsprachige Version des Working Alliance Inventory-short revised (WAI-SR)-Ein schulenübergreifendes, ökonomisches und empirisch validiertes Instrument zur Erfassung der therapeutischen Allianz (The German-Language Version of the Working Alliance Inventory-Short Revised (WAI-SR) - A Cross-School, Economic, and Empirically Validated Instrument to Capture the Therapeutic Alliance). *Klin Diagn Evaluation*, *1*(3), 343-358. <https://doi.org/10.7892/BORIS.27962>

Wolf, S., Seiffer, B., Zeibig, J.-M., Welkerling, J., Bauer, L. L., Frei, A. K., Studnitz, T., Rosenstiel, S., Fiedler, D. V., Helmhold, F., Ray, A., Herzog, E., Takano, K., Nakagawa, T., Kropp, S., Franke, S., Peters, S., El-Kurd, N., Zwanzleitner, L., . . . Ehring, T. (2021). Efficacy and Cost-Effectiveness of a Transdiagnostic Group-Based Exercise Intervention: Study Protocol for a Pragmatic Multi-Site Randomized Controlled Trial. *BMC psychiatry*, *21*(1), 540-556. <https://doi.org/10.1186/s12888-021-03541-3>

Wolf, S., Zeibig, J.-M., Hautzinger, M., & Sudeck, G. (2020). *Mental Health Through Exercise. ImPuls: an Exercise Intervention for Individuals with Psychiatric Disorders [Psychische Gesundheit durch Bewegung. ImPuls: Ein sport- und bewegungstherapeutisches Programm für Menschen mit psychischen Erkrankungen]* (first ed.). Julius Beltz GmbH & Co. KG.

Zabora, J., BrintzenhofeSzoc, K., Jacobsen, P., Curbow, B., Piantadosi, S., Hooker, C., Owens, A., & Derogatis, L. (2001). A new psychosocial screening instrument for use with cancer patients. *Psychosomatics*, *42*(3), 241-246. <https://doi.org/10.1176/appi.psy.42.3.241>

Zahra, D., Hedge, C. (2010). The Reliable Change Index: Why isn't it more popular in academic psychology? *PsyPag Quarterly*(76), 15.

Zeibig, J.-M., Seiffer, B., Frei, A. K., Takano, K., Sudeck, G., Rösel, I., Hautzinger, M., & Wolf, S. (2023). Long-term efficacy of exercise across diagnostically heterogenous mental disorders and the mediating role of affect regulation skills. *Psychol Sport Exerc*, *64*, 102340. <https://doi.org/10.1016/j.psychsport.2022.102340>

Zeibig, J.-M., Seiffer, B., Sudeck, G., Rösel, I., Hautzinger, M., & Wolf, S. (2021). Transdiagnostic Efficacy of a Group Exercise Intervention for Outpatients with Heterogenous Psychiatric Disorders: A Randomized Controlled Trial. *BMC psychiatry*, *21*(1), 313-330. <https://doi.org/10.1186/s12888-021-03307-x>

Zhang, C. Q., Zhang, R., Schwarzer, R., & Hagger, M. S. (2019). A meta-analysis of the health action process approach. *Health Psychol*, *38*(7), 623-637. <https://doi.org/10.1037/hea0000728>
